# Anti-aggregant tau mutant promotes neurogenesis

**DOI:** 10.1186/s13024-017-0230-8

**Published:** 2017-12-04

**Authors:** Maria Joseph, Marta Anglada-Huguet, Katharina Paesler, Eckhard Mandelkow, Eva-Maria Mandelkow

**Affiliations:** 1 German Center for Neurodegenerative Diseases (DZNE), Bonn, Germany; 2 CAESAR Research Center, Bonn, Germany; 3 Max-Planck-Institute for Metabolism Research, Cologne, Germany

**Keywords:** Tau, Hippocampus, CA3, Neurogenesis, Wnt

## Abstract

**Background:**

The microtubule-associated protein Tau plays a role in neurodegeneration as well as neurogenesis. Previous work has shown that the expression of the pro-aggregant mutant Tau repeat domain causes strong aggregation and pronounced neuronal loss in the hippocampus whereas the anti-aggregant form has no deleterious effects. These two proteins differ mainly in their propensity to form ß structure and hence to aggregate.

**Methods:**

To elucidate the basis of these contrasting effects, we analyzed organotypic hippocampal slice cultures (OHSCs) from transgenic mice expressing the repeat domain (RD) of Tau with the anti-aggregant mutation (Tau^RDΔKPP^) and compared them with slices containing pro-aggregant Tau^RDΔK^. Transgene expression in the hippocampus was monitored via a sensitive bioluminescence reporter gene assay (luciferase).

**Results:**

The expression of the anti-aggregant Tau^RDΔKPP^ leads to a larger volume of the hippocampus at a young age due to enhanced neurogenesis, resulting in an increase in neuronal number. There were no signs of activation of microglia and astrocytes, indicating the absence of an inflammatory reaction. Investigation of signaling pathways showed that Wnt-5a was strongly decreased whereas Wnt3 was increased. A pronounced increase in hippocampal stem cell proliferation (seen by BrdU) was observed as early as P8, in the CA regions where neurogenesis is normally not observed. The increase in neurons persisted up to 16 months of age.

**Conclusion:**

The data suggest that the expression of anti-aggregant Tau^RDΔKPP^ enhances hippocampal neurogenesis mediated by the canonical Wnt signaling pathway, without an inflammatory reaction. This study points to a role of tau in brain development and neurogenesis, in contrast to its detrimental role in neurodegeneration at later age.

**Electronic supplementary material:**

The online version of this article (10.1186/s13024-017-0230-8) contains supplementary material, which is available to authorized users.

## Background

Tau plays an important role in brain development and neurogenesis. The 3-repeat fetal isoform of Tau is expressed in newborn granule cells [26], whereas 4-repeat adult Tau isoforms are involved in promoting neuronal differentiation, neurite and axonal outgrowth [14, 67]. The phosphorylation of Tau is developmentally regulated, as it is higher in the fetal neurons and decreases with their maturation [10]. In many neurodegenerative diseases Tau forms neurofibrillary tangles, even though it is a highly soluble natively unfolded protein [81]. The aggregation of Tau is based on its repeat domain that harbors two hexapeptide motifs with a propensity for β-structure which is responsible for Tau aggregation and pathology [80]. Based on the structural analysis and cell models, our laboratory generated transgenic mouse models to study the influence of Tau aggregation on pathology. One of the models expresses the 4R-repeat domain, combined with the aggregation-promoting gene mutation ∆K280 observed in cases of Frontotemporal Dementia (FTD) and Alzheimer Disease (AD) [57, 65]. These mice showed extensive Tau missorting, aggregation, astrogliosis, loss of synapses, and neurodegeneration [56, 72]. By contrast, an "anti-aggregant" mouse line was generated, expressing the 4R tau repeat domain with the same ∆K280 mutation but with two additional proline mutations to neutralize the ß-sheet formation. In this line the aggregation is suppressed, and the mice do not show cognitive deficits even at advanced age. These findings supported the view that pathological effects of Tau depend on its ability to aggregate.

Another initial assumption was that the anti-aggregant Tau did not perturb the normal functions of Tau, particularly since the expression of the Tau repeat domain was low and therefore had only minimal effects on microtubule stability. However, in order to test this, we investigated this mouse line in more detail using organotypic hippocampal slices. This revealed the surprising result that anti-aggregant Tau is not simply a passive bystander, but actively promotes neurogenesis. This is reminiscent of other reports showing that neurogenesis plays an important role in the pathophysiology of neurodegenerative Tauopathies like AD, PD, or FTD [48, 73, 82, 83]. Pathological forms of Tau cause defects in neurogenesis and neural plasticity that precede the aggregation of Tau and Aß [22, 29, 44]. A decline in neurogenesis correlates with an increased release of CSF tau [41], an early marker for neurodegeneration. Regenerative strategies to enhance the endogenous production of the neuronal progenitor cells from the NSCs [15], induced by drugs [8, 52], physical exercise [16, 76, 77] or environmental enrichment [39, 40] have been proposed as therapeutic approaches. Conversely, this implies that anti-aggregant forms of Tau could protect against neurodegeneration by boosting neurogenesis, as reported here.

## Methods

Transgenic mice expressing the human Tau four-repeat domain with the aggregation-promoting FDTP-17 mutation ∆K280 (Tau^RD∆K^, 129 residues, M-Q244-E372 without K280) or with the additional anti-aggregant Ile277Pro and Ile208Pro mutations (Tau^RD∆KPP^) and reporter gene firefly luciferase under control of a Tet-operon response element (tetO) [56], were crossed with CaMKIIα-tTA mice (Mayford et al, 1996) to generate a regulatable Tet-off system. Animals were housed and tested according to standards of the German Animal Welfare Act.

### Assessment of luciferase activity

Since the mouse lines were generated using a bidirectional promoter to express both the Tau construct (pro-aggregant Tau^RD∆K^ or anti-aggregant Tau^RD∆KPP^) and the reporter protein firefly luciferase, its enzymatic activity can be used to quantify the expression levels and regional distribution (Contag, 2007). Photon emission was detected with a luminometer (IVIS Spectrum) at 560-660 nm (Caliper Life Sciences, Germany) after incubation of cultures with 470 μM of the luciferase substrate D-luciferin (Caliper Life Sciences, Germany) and measured after 10, 15, 20, 25 and 30 days in culture.

Hippocampal organotypic slice cultures were prepared following Stoppini et al. [69], with modifications. Briefly, 8-10 days old mice were decapitated, brains were rapidly removed and hippocampi dissected at 4°C. A McIIwain tissue chopper (Gabler, Bad Schwabach; Germany) was used to prepare 400 μm thick transverse slices which were transferred to semi-porous cell culture inserts (Millipore, Bedford, MA, 0.4 μm). Inserts containing 6-8 slices were placed in six well culture trays containing 1 ml of culture media (50% MEM, 25% HBSS, penicillin/streptomycin (all from PAA, Austria), 25% horse serum, 4.5 mg/ml glucose (Sigma, Germany), pH 7.4). The culture medium was changed on the first day after preparation and afterwards every 3^rd^ day. Slices were kept in culture for 3 - 4 weeks. Suppression of the human Tau transgene was achieved by adding doxycycline (Sigma, Germany) to the culture media (final concentration 2 μg/ml). During treatment, doxycycline was refreshed every 3^rd^ day together with full medium change.

### BrdU labeling in organotypic hippocampal slice cultures

Slices were cultured under the normal culturing conditions until DIV15. From DIV15 onwards, 50μM of BrdU was dissolved in the normal slice culture media and the slices were cultured until DIV30. BrdU containing culture media was refreshed during every culture media change. At DIV30, the slices were fixed and used for further immunohistochemical analysis.

### Biochemistry of slice cultures

To estimate protein expression, cultured hippocampal slices (6–8, prepared and pooled from the same animal) were homogenized in lysis buffer [50 mM Tris-HCl, pH 7.4, 10% glycerol, 1% NP-40, 5 mM DTT, 1 mM EGTA, 20 mM NaF, 1 mM Na_3_VO_4_, 150 mM NaCl, protease inhibitors (Complete Mini; Roche, Indianapolis, IN), 5 mM CHAPS (3-[(3-cholamidopropyl)dimethylammonio]-1-propanesulfonate), 100 U/ml benzonase, 5 μm okadaic acid]. Slice homogenates were resolved by SDS-PAGE (10% or 17% polyacrylamide gels) and transferred to polyvinylidene fluoride (PVDF) membranes (Millipore, Bedford, MA). The membrane was incubated in 5% non-fat milk in TBS-Tween for 1 h at RT, washed with TBS-Tween the next day and incubated overnight in primary antibody solution at 4°C. The membrane was washed with TBS-Tween and incubated with the secondary antibody (Dako, Germany) coupled to horseradish peroxidase (HRP) for 1 h at RT. The membrane was developed by ECL Western Blotting Detection Kit (GE Healthcare, USA) and analyzed by densitometry (LAS 3000; AIDA software; Raytest, Straubenhardt, Germany). The following antibodies were used: pan Tau antibody (K9JA) (DakoCytomation, Carpinteria, CA) (1:5000); anti-GFAP antibody (Cell signaling technology) (1:1000), anti-Wnt3 antibody (GenTex) (1:500); anti-Wnt5a antibody (Abcam) (1:500), total-GSK3ß (Sigma, Germany) (1:1000), pGSK3ß(Y216) (Sigma, Germany) (1:1000) anti-ß actin (Sigma, Germany) (1:10.000) and secondary antibodies, HRP-anti-rabbit and HRP-anti-mouse (DakoCytomation, Carpinteria, CA).

### Immunohistochemistry on organotypic hippocampal slice cultures

Slice cultures were left attached on the Millicell membrane and stained as free-floating sections in 6-well plates. Cultures were first fixed with 4% paraformaldehyde in PBS (PAA, Austria) for 2 h at 4°C. After washing with cold PBS, slices were permeabilized by 0.4% TritonX-100/PBS for 90 min at RT. Slices were then blocked with 5% BSA for 2 h and afterwards incubated with primary antibody diluted in PBS for 2-3 days at 4°C. After washing with PBS, slices were incubated with secondary antibody for 2 days at 4°C. After washing, slices were mounted with Permafluor mounting solution (Beckman Coulter, Paris, France), cover-slipped and dried before imaging. The following primary antibodies were used: monoclonal anti-neuronal nuclei (NeuN) antibody (Chemicon International, Temecula, CA) (1:1000), pan-Tau antibody K9JA (Dako, Hamburg, Germany, Nr. A0024 (1:1000)); anti-Iba1 antibody (Wako Chemicals, Germany) (1:1000); anti-GFAP antibody (Cell signaling technology) (1:1000), anti-BrdU (GenTex) (1:1000), DCX (Cell Signalling) (1:500) and Ki67 (Abcam) (1:500). All fluorescent (goat anti-rabbit/mouse cyanine 2, 3 and 5)-labeled secondary antibodies were from Dianova (Hamburg, Germany) (1:1000).

### BrdU injection in P8 pups

Pups at post-natal day 8, from both the control and the anti-aggregant groups were taken. Their body weight and gender was noted. BrdU (Sigma Aldrich) was dissolved in 0.9% saline and 50mg/kg body weight was injected into each animal carefully. The pups were kept alive for 2 hours after the BrdU injection then killed by cervical decapitation and their brains removed for further immunohistochemical analysis.

### Immunohistochemistry on free floating sections

Post-natal brain from P8 animals and brain of the 2 month old animals were removed by cervical decapitation and immediately fixed in 4%PFA solution for over 2 days at 4°C. The brain was then cut into 40μm thick coronal free floating sections with the help of a vibratome at RT. For immunofluorescence staining the sections were then collected in the preservation solution (0.1M PBS+ 30% Sucrose) until further use. The sections were first permeabilized with 0.4% Triton X-100 followed by the antigen retrieval step, where the free floating sections were heated in pre-heated sodium citrate buffer at 80°C for 30 min. The sections were then kept in the same sodium citrate buffer until the temperature of the buffer cools down to RT, washed with PBS and blocked with 5% horse serum for a minimum of 2 hours. Primary antibodies were then diluted in PBS and the free floating sections were incubated overnight at 4°C followed by the secondary antibody either overnight or for a minimum of 2 hours.

### Microscopy

Images were acquired with an Olympus laser scanning microscope FV1000 (Olympus, Tokyo), equipped with confocal laser scanning unit, argon (Ar; 488 nm) and helium/neon (He/Ne 543 nm and 633 nm). For 2 or 3 channel imaging, images were acquired via sequential scanning. Image stacks were collected for the whole hippocampus at lower magnification and for all hippocampal subfields at higher magnifications. Digital zoom was used for fluorescent dye tracing of single neurons and spines. Maximum projection images were generated from resulting *Z* stacks using ImageJ software (NIH).

### Stereology

Volume estimation using the Cavalieri principle on transgenic and control mice was performed with stereo investigator software (MBF Biosciences) driving a motorized stage. Cavalieri analysis was performed with the investigator blind to genotype. To determine the number of neurons in the different areas of the hippocampus, unbiased counting was performed with stereo investigator software. The brains were dissected and fixed immediately with 4%PFA for 72 hours, then changes to 30% sucrose in PBS. Then the brains were cut into 40μm thick free-floating sections. Every 8^th^ section of the hippocampus was immunostained with NeuN and then volume analysis was done on these sections. The volume of the hippocampus thus obtained is quoted as the apparent volume, without correction for shrinkage during brain slice preparation process.

### Statistical analysis

All data are expressed as mean ± SEM. All graphs were created with GraphPad Prism 7. Different statistical analyses were performed as appropriate, and indicated in the figure legends. Briefly, experimental data were analyzed either by Student's t-test (when only one parameter was analyzed). Data are shown as mean ± SEM. p values are as follows: *p < 0.05, **< 0.01, and ***p < 0.001 ***p<0.001.

## Results

### Expression and distribution of Tau repeat domain (Tau^RD^) constructs

**Fig. 1:**
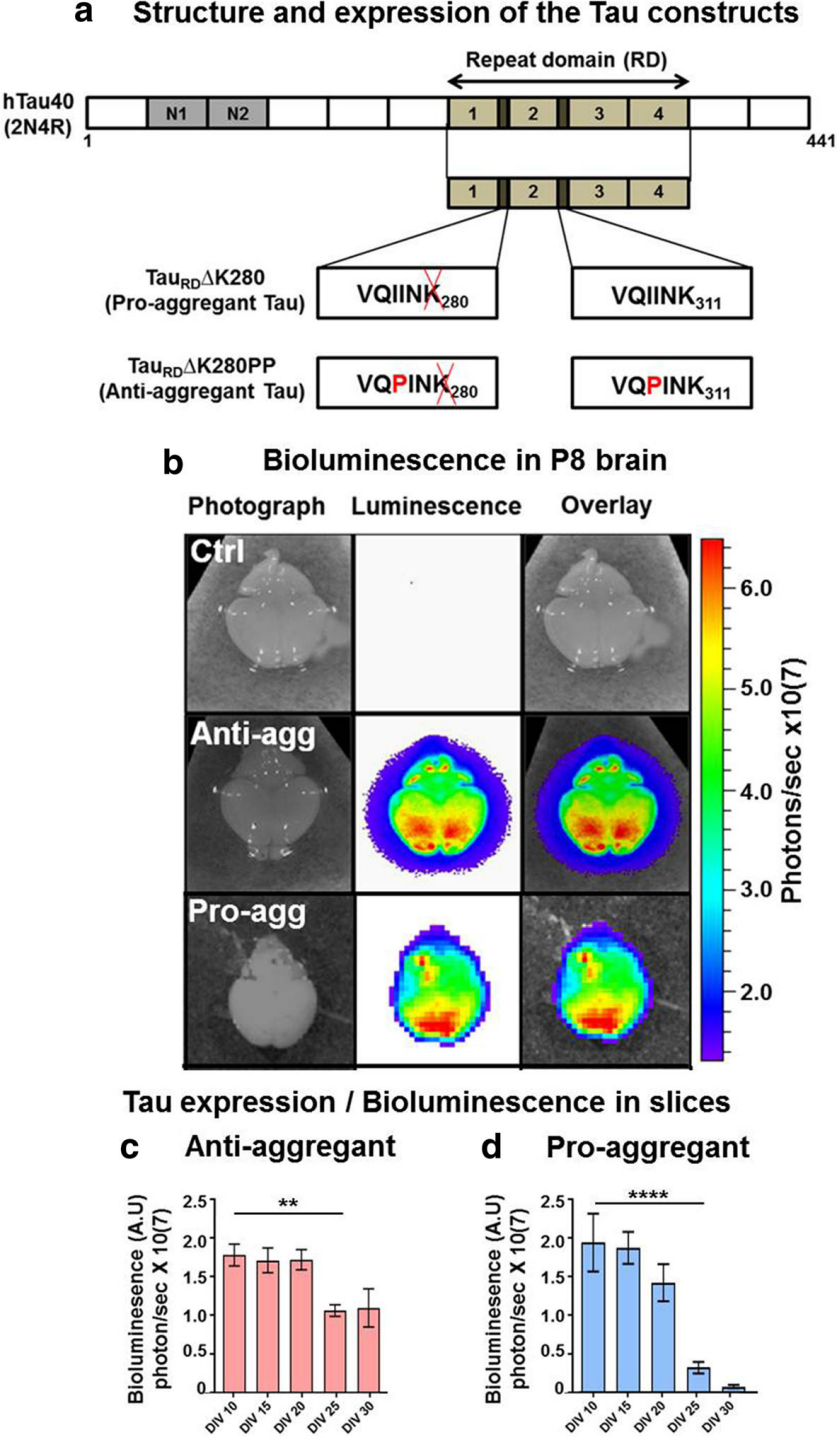
Expression level of the exogenous human anti-aggregant Tau^RDΔKPP^ and pro-aggregant Tau^RD∆K^. **a** The anti-aggregant and pro-aggregant construct is based on the human Tau 4-repeat domain carrying the FTDP-17 mutation ΔK280 near the beginning of R2. The four repeats in the C-terminal half of Tau are highlighted in green (R1-R4). The two hexapeptide motifs at the beginning of R2 and R3 are capable of promoting aggregation by inducing ß-structure. The ΔK280 mutation promotes aggregation, and the two proline mutations inhibit it. **b** Luciferase activity within the brains of pups: Control (top row), expressing anti-aggregant Tau^RDΔKPP^ (middle row) or pro-aggregant Tau^RDΔK^ (bottom row) at post-natal day 8 (P8), as determined by bioluminescence (photons/sec). The control brain does not show any luciferase signal, the anti-aggregant Tau^RDΔKPP^ brain shows an intense luciferase signal (middle and right panel), ranging from 2 - 6x 10^7^ photons per second (blue to red, see heat scale on right), predominantly in the frontal part of the brain. A similar expression pattern is seen in the pro-aggregant Tau^RDΔK^ brain at P8. **c** Luciferase activity monitored in slices of anti-aggregant mice from DIV10 to DIV30. The signal remains roughly constant from DIV10 to DIV20 and then declines down to ~40% at DIV25-30 (because of the aging process in the slice cultures). Data are expressed as a mean ±SEM of 4-6 slices from 10 different animals and analyzed by unpaired Student´s t-test. ** *P*-value <0.01. **d** Luciferase activity monitored in slices of pro-aggregant mice from DIV10 to DIV30. There is a similar nearly constant expression of Tau^RD∆K^ from DIV10-20, followed by a strong decline. Data are expressed as a mean ±SEM of 4-6 slices from 10 different animals and analyzed by unpaired Student´s t-test. **** *P*-value <0.0001

Figure 1a depicts the human Tau variants used in generating the transgenic mouse lines. They are expressed together with a luciferase reporter on the same plasmid under control of a bidirectional transactivator (tTA)-responsive promoter, combined with forebrain-specific expression of tTA under the CaMKIIα promoter. In this tet-off system, gene expression in the forebrain can be switched off or on, respectively, by addition or removal of doxycycline, both in mice and organotypic slices [33, 55]. This allows one to monitor the expression of Tau via the bioluminescence of luciferin in living mice and in slices, using the IVIS in-vivo imaging system.

P8 animals were used for the preparation of organotypic hippocampal slice cultures (OHSCs), and the expression level of anti-aggregant Tau^RDΔKPP^ and pro-aggregant Tau^RDΔK^ pups at P8 was measured by bioluminescence (Fig. 1b). The brains from the transgenic pups showed enhanced bioluminescence in the range of 2.0-6.0x 10^7^ photons/second (after application of 5μl of 2μM luciferin on the dissected brain). The expression was high in the forebrain region (as expected for the CaMKIIα promoter, Fig. 1b, red color). OHSCs were cultured under optimal conditions up to 30 days in vitro (DIV30).

For analyzing the expression of anti-aggregant Tau^RDΔKPP^ and pro-aggregant Tau^RDΔK^ in slice cultures, we directly applied luciferin into the culture media and the bioluminescence was measured by IVIS over the whole culturing period. In the anti-aggregant slices the bioluminescence signal remains roughly constant from DIV10 until DIV20, then decreases down to ~40% of the value at DIV25 (Fig. 1c). In the pro-aggregant slices the expression was similar to the anti-aggregant Tau^RDΔKPP^ at DIV10-15, but then declines rapidly (Fig. 1d). This may be due to the strong Tau pathology of the toxic pro-aggregant Tau^RDΔK^ leading to Tau aggregation, loss of synapses and loss of neurons. A similar decrease of the bioluminescence signal is observed in live animals (both anti-aggregant and pro-aggregant mice), except that it takes place on a ~5-fold longer time scale, i.e. high expression during the first two post-natal months [32].

### Expression of anti-aggregant Tau^RDΔKPP^ leads to an increase in neurons

**Fig. 2:**
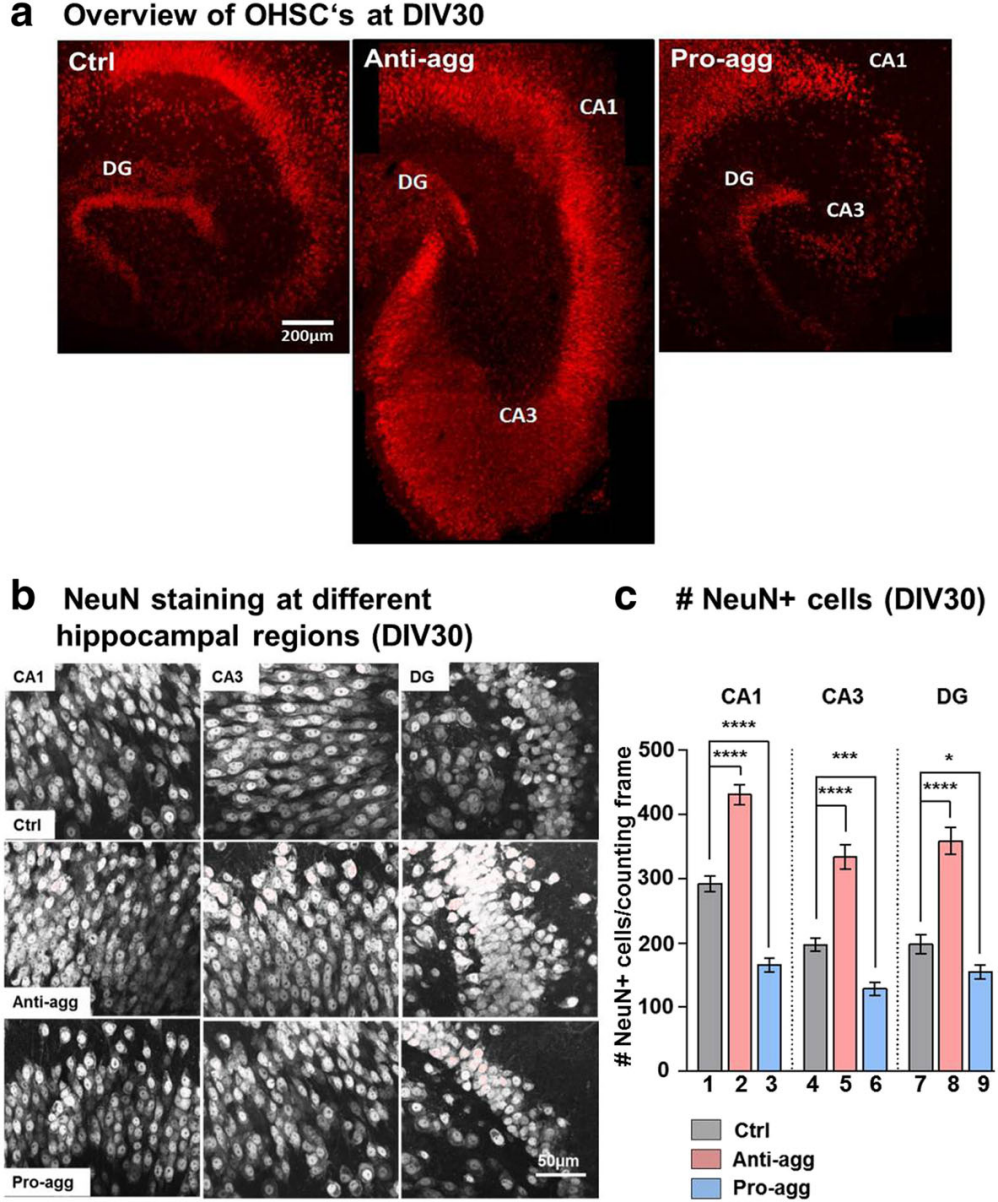
Increased number of neurons in anti-aggregant Tau^RDΔKPP^ organotypic hippocampal slice culture. **a** OHSCs were prepared from control, anti-aggregant Tau^RDΔKPP^ and pro-aggregant Tau^RDΔK^ pups aged P8. Slices were fixed at DIV30 and immunostained with NeuN antibody. Overview image of a single slice aged DIV30 from the controls, anti-aggregant Tau^RDΔKPP^ and the pro-aggregant Tau^RDΔK^ shows a 30% increase of hippocampal size in the anti-aggregant Tau^RDΔKPP^ slice compared to the age-matched controls. On the contrary the pro-aggregant Tau^RDΔK^ slice shows a similar size of the hippocampus as in the controls but with intense neuronal loss in the CA3 region. Scale bar 200μm. **b** Representative image of the CA1, CA3 and DG regions of the hippocampus stained with NeuN from controls, anti-aggregant Tau^RDΔKPP^ and pro-aggregant Tau^RDΔK^ slices. Scale bar 50μm. Cell bodies of pyramidal neurons in the CA region and the granular neurons in the DG region are clearly distinguishable. **c** Graph showing the number of NeuN^+^ cells per counting frame in the different regions of the hippocampus. An increase by 47% of the number of NeuN^+^ cells is observed in the CA1, 69% in CA3 and 81% increase in neuronal number in DG in the anti-aggregant Tau^RDΔKPP^ slices (pink bars) when compared to the controls (grey bars). By contrast, the pro-aggregant Tau^RDΔK^ slices (blue bars) show 44% reduced neuronal number in the CA1, 33% reduced neuronal number in CA3 and 22% DG compared to the age matched controls (grey). Results are given as mean ±SEM of 10 animals and 4-6 slices per animal. Data were analyzed by Student's t-test. *p<0.05, ***p<0.001 ****p<0.0001 compared to control slices

OHSCs at DIV30 were fixed and immunostained for NeuN, a marker for mature neurons. Microscopic analysis revealed a remarkable overall increase (30%) in the size of the hippocampus of anti-aggregant Tau^RDΔKPP^ OHSCs, compared to controls and the age-matched pro-aggregant Tau^RDΔK^ slices (Fig. 2a). To verify whether this was due to an increase in neuronal number, individual regions of the hippocampus were imaged (Fig. 2b) and neuronal numbers counted manually using ImageJ software. This revealed an impressive increase in mature neurons in all regions of the hippocampus (47% in CA1; 69% in CA3 and 81% in DG) in the anti-aggregant Tau^RDΔKPP^ slices compared to age-matched controls (Fig. 2c, bars 2, 5 and 8). By contrast, the pro-aggregant Tau^RDΔK^ slices showed a pronounced reduction in neuronal numbers, particularly in the CA1 (-44%), CA3 (-33%) and DG (-22%) regions compared to controls (Fig. 2c, bars 3, 6 and 9). Thus, pro-aggregant Tau^RDΔK^ causes neurodegeneration, whereas anti-aggregant Tau^RDΔKPP^ leads to neurogenesis, even in regions outside the DG.

### Decrease of microglia in anti-aggregant Tau^RDΔKPP^ slices

**Fig. 3:**
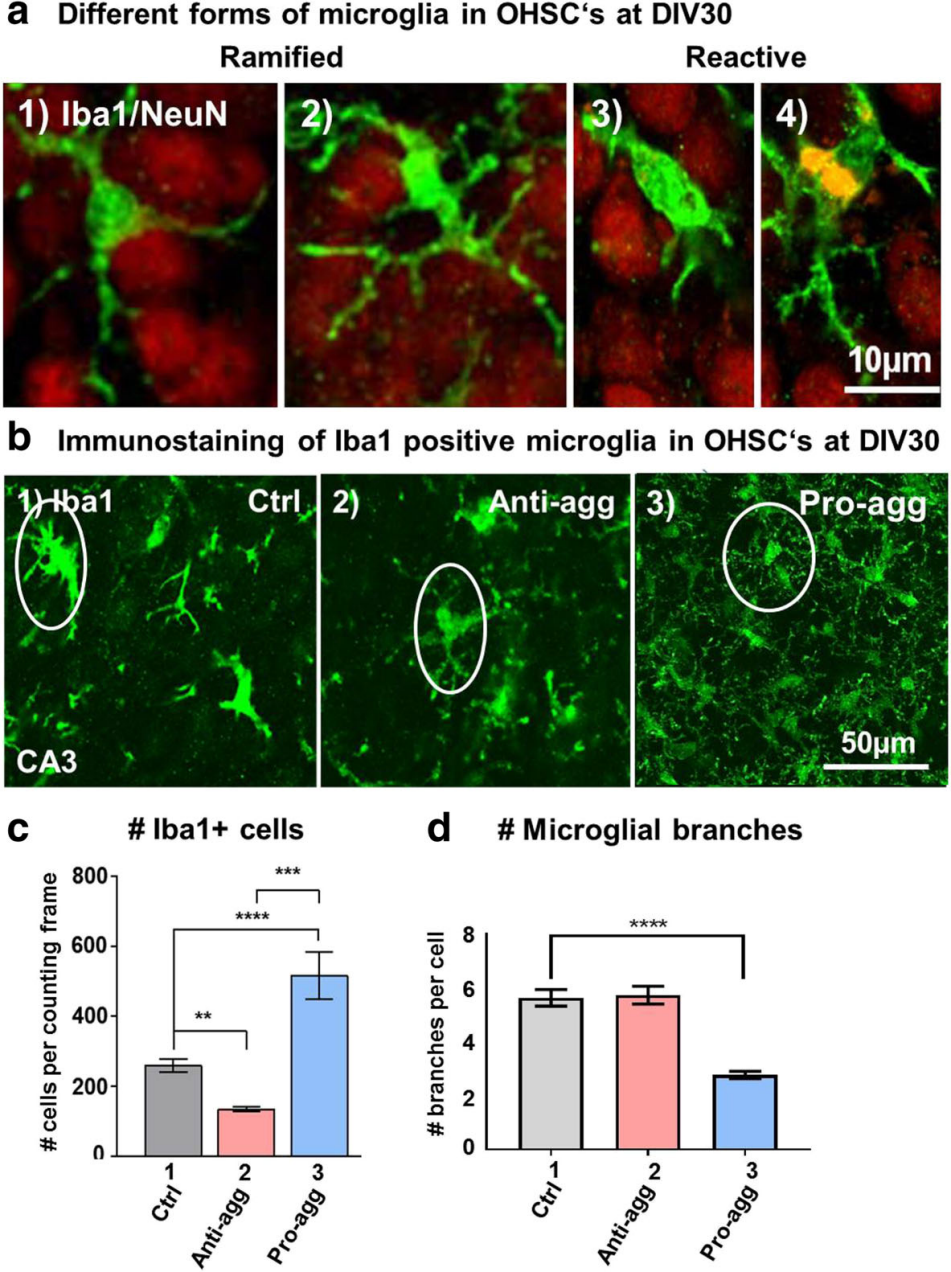
Decrease in microglial number in anti-aggregant Tau^RDΔKPP^ slices and increased microgliosis in the pro-aggregant Tau^RDΔK^ slices. OHSCs from control, anti-aggregant Tau^RDΔKPP^ and pro-aggregant Tau^RDΔK^ P8 pups were prepared and cultured until DIV30. Slices were stained with Iba1 antibody against microglia. **a** Representation of different types of microglia in the OHSCs. OHSCs from the control slices at DIV30 were immunostained with Iba1 (green) for microglia and NeuN (red) for neurons. The two panels on the left show the ramified form of microglia with multiple branches. The two right panels show the reactive form of microglia with fewer processes. Interestingly the last panel shows a microglia engulfing neuronal debris (note that the debris is stained red but appears yellow in the co-localization image). Scale bars = 10μm in all panels. **b** Overview of Iba1 staining of microglia (green) in the CA3 hippocampal region of control, anti-aggregant Tau^RDΔKPP^ and pro-aggregant Tau^RDΔK^ slices (scale bar 50μm). The controls and anti-aggregant Tau^RDΔKPP^ slices show ramified morphology of microglia whereas the pro-aggregant Tau^RDΔK^ slices had the reactive form of microglia. **c** Quantification of the number of Iba1 positive microglial cells in control, anti-aggregant Tau^RDΔKPP^ and pro-aggregant Tau^RDΔK^ slices at DIV30. All the Iba1 positive microglia were counted manually (as circled in the figure) by ImageJ. There was a reduction by 50% in the number of microglia in the anti-aggregant Tau^RDΔKPP^ slices at DIV30 compared to age-matched controls. By contrast, pro-aggregant Tau^RDΔK^ slices showed a massive increase of up to 100% in microglial number. Results are given as mean ±SEM of 10 animals and 4-6 slices per animal. Data were analyzed by Student's t-test., **p<0.01, ***p<0.001 and ****p<0.0001. **d** Quantification of the number of branches in microglia in control, anti-aggregant Tau^RDΔKPP^ and pro-aggregant Tau^RDΔK^ slices at DIV30. The branches were counted manually using the ImageJ software. The controls and the anti-aggregant Tau^RDΔKPP^ microglia showed 6-7 branches on average. By contrast, the pro-aggregant Tau^RDΔK^ microglia showed only 2-3 branches compared to the age-matched controls. Results are given as mean ±SEM of 10 animals and 4-6 slices per animal. Data were analyzed by Student's t-test ****p<0.0001 compared to control slices

To study the effect of anti-aggregant Tau^RDΔKPP^ and pro-aggregant Tau^RDΔK^ on glial cells, we investigated the status of microglial morphology and number in the slice cultures. OHSCs at DIV30 were immunostained for neurons (marker NeuN, Fig. 3a; red) and for microglia (marker Iba1; Fig. 3a; green). The ramified/active microglia (with many (6-7) and longer processes, typically 5-10 μm) and rounded/reactive microglia (fewer (2-3) and shorter processes ≤ 5μm) are clearly distinguishable in the slice cultures. In controls and anti-aggregant Tau^RDΔKPP^ slices, the microglia were mainly in the ramified form, in contrast to the pro-aggregant Tau^RDΔK^ slices where microglia were more of the reactive form (Fig 3b). Total numbers of Iba1 positive microglial cells were reduced to 50% in anti-aggregant Tau^RDΔKPP^ slices when compared to controls (Fig. 3c, bar 2). The opposite result was observed in the pro-aggregant Tau^RDΔK^ slices where the microglial number was increased by 100% compared to controls (Fig. 3c, bar 3). Since the number of microglia is inversely correlated with the number of stem/progenitor cells in the granular cell layer [28] this data argues that neurogenesis is enhanced in the anti-aggregant Tau^RDΔKPP^ slices.

We further investigated the number of branches in microglia, aiming to address the activation status of microglial cells in slice cultures. The branch number was counted manually with ImageJ software. The anti-aggregant Tau^RDΔKPP^ slices had ramified form of microglia with 6-7 branches on an average indicating that the microglial cells were in their normal physiologically active form and there is no sign of inflammation (Fig 3d, bars 1 and 2). On the contrary, microglia in pro-aggregant Tau^RDΔK^ slices, were increased in number and were also observed with 2-3 branches on an average compared to age-matched controls and also the anti-aggregant Tau^RDΔKPP^ slices (Fig. 3d, bar 3). This indicates that in the pro-aggregant Tau^RDΔK^ slices the microglia are in a reactive form, indicating that there is also enhanced inflammation.

### Anti-aggregant Tau^RDΔKPP^ slices show reduced age-related hypertrophy of astrocytes

**Fig. 4:**
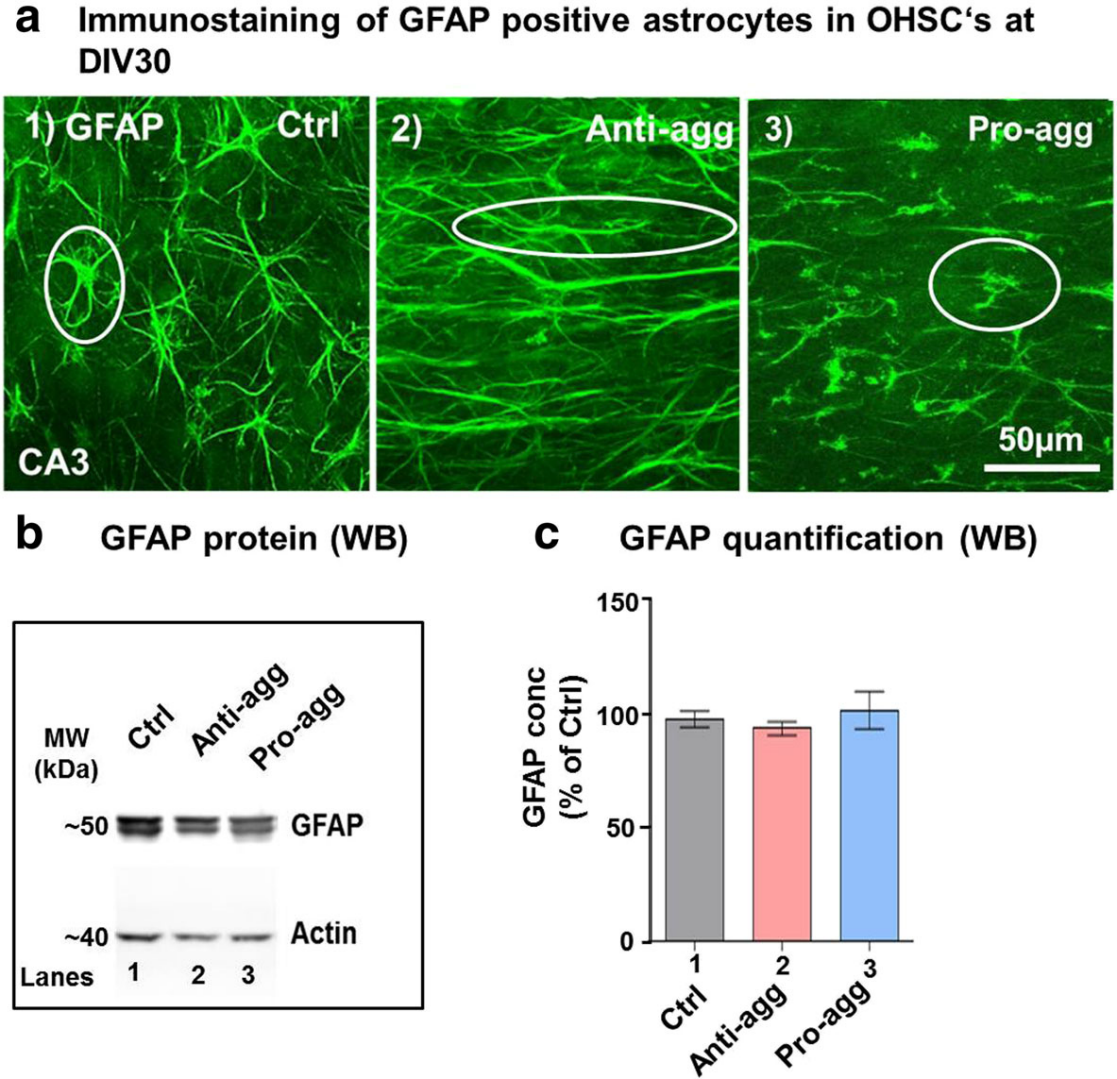
Decrease of hypertrophic astrocytes in anti-aggregant Tau^RDΔKPP^ slices but no changes in the global GFAP content. **a** OHSCs from controls, anti-aggregant Tau^RDΔKPP^ and pro-aggregant Tau^RDΔK^ P8 pups were prepared and cultured until DIV30. Slices were stained with GFAP antibody (green) to label astrocytes. The control slices at DIV30 showed star-shaped astrocytes (left). In anti-aggregant Tau^RDΔKPP^ slices the astrocytes have smaller cell bodies and long, thin processes (middle). By contrast, pro-aggregant Tau^RDΔK^ slices showed hypertrophic astrocytes (right) with fewer processes which are typical of neurodegeneration. **b** Biochemical analysis of GFAP protein levels. Protein extracts from OHSCs of control, anti-aggregant Tau^RDΔKPP^ and pro-aggregant Tau^RDΔK^ slices at DIV30 were subjected to western blot (loading 3μg/lane) to analyze for GFAP protein levels. **c** Quantification of the western blots showing that the GFAP protein contents of the three samples are similar. Results are given as mean ±SEM of 4-6 slices per mice and 5-6 mice and represent the ratio between GFAP and actin levels. Data were analyzed by Student's t-test

Astrocytes are needed for normal neurotransmission, to take up glutamate, reduce excitotoxicity [2] and to regulate the ionic microenvironment in the brain. Morphological changes in astrocytes are related to brain plasticity, synaptogenesis and aging. In particular, hypertrophic astrocytes (with more thickened processes) are increased in the aging brain [79]. OHSCs from control, anti-aggregant Tau^RDΔKPP^ and pro-aggregant Tau^RDΔK^ slices were fixed at DIV30 and immunostained for astrocytes with GFAP antibody (Fig. 4a). The control slices had star-shaped astrocytes typical of the normal physiological forms having prominent cell bodies with 8-10 processes ranging from 6-10μm in length (Fig. 4a1). The anti-aggregant Tau^RDΔKPP^ slices had smaller cell bodies with thin, long and slender branches (Fig. 4a2). Such astrocytes are thought to be related to maturation of synapses between hippocampal neurons [11]. On the contrary the pro-aggregant Tau^RDΔK^ slices showed hypertrophic astrocytes with prominent cell bodies with 1-3 processes ranging from 3-5μm in length, which can be compared to the degrading forms of astrocytes (Fig. 4a3). Because of these morphological differences in the GFAP immunostaining and the ambiguities in counting astrocyte cell numbers we further checked for GFAP protein levels by western blotting. The OHSCs at DIV30 were collected and homogenized for biochemical protein analysis by a GFAP antibody, revealing that the GFAP protein content was roughly comparable in the controls, anti-aggregant Tau^RDΔKPP^ and pro-aggregant Tau^RDΔK^ slices (Fig 4b and c).

### Endogenous mouse Tau is increased in anti-aggregant Tau^RDΔKPP^ slices

**Fig. 5:**
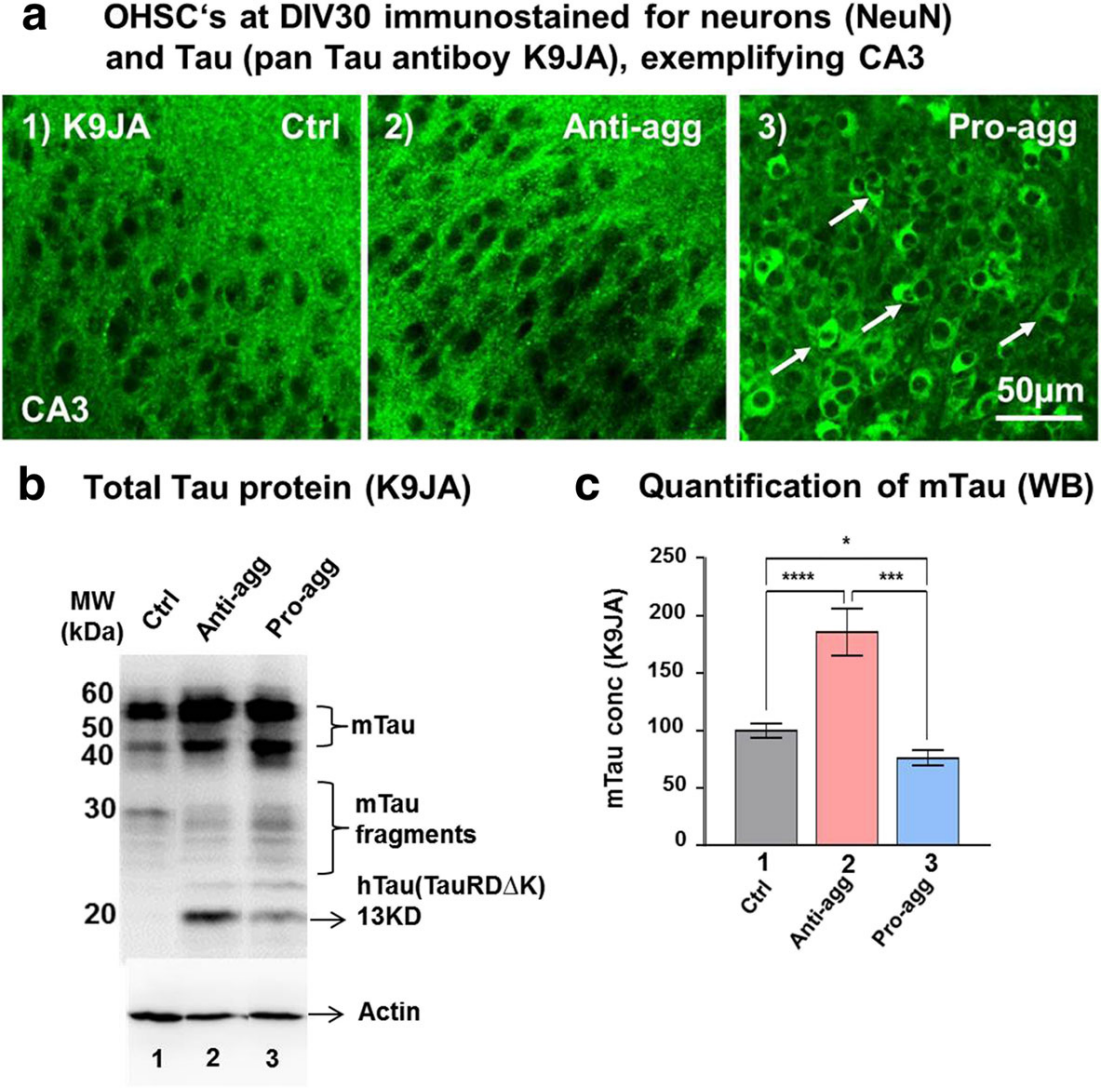
Increased endogenous Tau in anti-aggregant Tau^RD∆KPP^ slices at DIV30. **a** OHSCs from control, anti-aggregant Tau^RDΔKPP^ and pro-aggregant Tau^RDΔK^ P8 pups were cultured until DIV30. Slices were stained with K9JA (pan Tau antibody) for the distribution of Tau. The control and the anti-aggregant Tau^RDΔKPP^ slices show uniform axonal distribution of Tau as detected by K9JA. By contrast the pro-aggregant Tau^RDΔK^ slice shows mislocalization of Tau into the somato-dendritic compartment, most prominently in the CA3 region (arrows). **b** Tau levels at DIV30: Protein extracts from OHSCs of controls, anti-aggregant Tau^RDΔKPP^ and pro-aggregant Tau^RDΔK^ slices were analyzed by western blotting using Tau antibody K9JA (loading 3μg/lane). **c** Quantification of the western blot showed a 80%increase in the amount of mouse Tau (by K9JA staining) in the anti-aggregant Tau ^RD∆KPP^ slice cultures when compared to the controls. There was a 20% reduction in the amount of Tau observed between the pro-aggregant Tau ^RD∆K^ and the control slices. Results are given as mean ±SEM of 10 animals and 3 slices per animal and represent the ratio between immunolabelled Tau and actin levels, obtained by densitometry analysis of western blots. Data were analyzed by Student's t test. *p<0.05, ***p<0.001 and ****p<0.0001 compared to control slices

OHSCs from controls, anti and pro-aggregant slices at DIV30 were fixed and immunostained for K9JA (a pan Tau polyclonal antibody) in order to observe the localization of Tau (Fig. 5a). In the controls and the anti-aggregant Tau^RDΔKPP^ slices there was a uniform axonal distribution of Tau in the hippocampus (Fig. 5, A1 and 2). The pro-aggregant Tau^RDΔK^ slices showed intense mislocalization of Tau in the somato-dendritic compartment, the most affected being the CA3 pyramidal neurons (Fig. 5, A3). We further checked for the total Tau levels in all the groups. Endogenous mouse Tau (comprising mainly the 4R isoforms) runs at a molecular weight of ~50-60 kDa, whereas the exogenous human anti-aggregant Tau^RDΔKPP^ and the pro-aggregant Tau^RDΔK^ run at 13 kD. Surprisingly, there was an ~80% increase in the endogenous mouse Tau level in slices obtained from pups expressing anti-aggregant Tau^RDΔKPP^ (Fig. 5b lane 2 and 5c bar 2), compared with age matched non-transgenic controls and pro-aggregant Tau^RDΔK^ (Fig. 5b lane 1 and 3; and 5c lane 1 and 3). In spite of this increase, there was little mislocalization of Tau (endogenous or exogenous) into the somato-dendritic compartment in the anti-aggregant Tau^RDΔKPP^ (Fig. 5, A2). By contrast, pro-aggregant Tau^RDΔK^ slices showed no overall increase in endogenous mouse Tau (rather a 20% decrease), yet pronounced mislocalization (Fig. 5, A3).

To confirm that the increase in endogenous mouse Tau was due to the expression of the anti-aggregant Tau, we further performed the switch-off experiments in the anti-aggregant Tau^RDΔKPP^ slices. From DIV15 until DIV30 the anti-aggregant slices were treated with DOX to switch off the expression of the anti-aggregant Tau. This lead to a strong reduction (~70%) of the endogenous mouse Tau (Fig. 10c, lane3). Since GSK3ß is a major Tau kinase involved in Tau phosphorylation and neurogenesis [34] we checked for the total levels of GSK3ß and its active form in brain lysates. However, there was no significant difference between controls and anti-aggregant mice (Additional file 1: Figure S2B).

### Hippocampal stem cell proliferation increases in anti-aggregant Tau^RDΔKPP^ slices

**Fig. 6:**
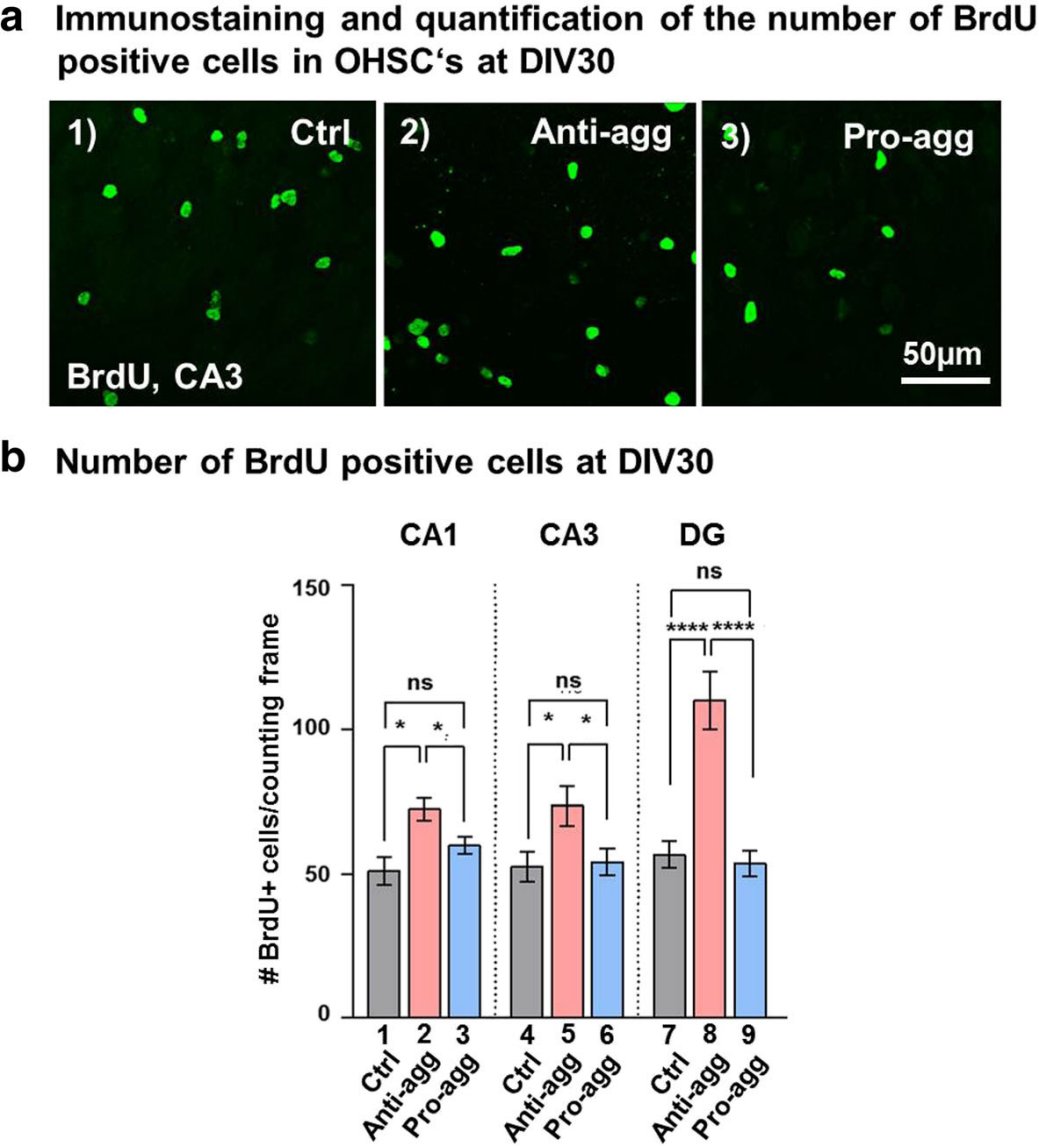
Proliferation assay in slice cultures. BrdU (50μM) was applied from DIV15 of the culturing period until DIV30 and refreshed at every culture media change. The slices were fixed with 4% formaldehyde and immunostained by anti-BrdU antibody. **a** Representative image of BrdU positive cells in the hippocampal CA3 region in the controls, anti-aggregant Tau^RDΔKPP^ and pro-aggregant Tau^RDΔK^ slices. BrdU positive proliferating cells were observed in the CA1, CA3 and the DG regions of the hippocampal slice cultures. The localization of these proliferating cells is not only restricted to the SGZ of the DG in OHSCs. **b** Quantification of the number of BrdU positive cells in the CA1, CA3 and the DG regions in the controls, anti-aggregant Tau^RDΔKPP^ and pro-aggregant Tau^RDΔK^ slices. There is an increase in BrdU positive proliferating cells by 30% in CA1 and CA3 and 100% increase in DG region in the anti-aggregant Tau^RDΔKPP^ slices (pink bars). Note that the pro-aggregant Tau^RDΔK^ slices do not show a significant difference in the number of BrdU positive cells (blue bars) compared to that of the controls (grey bars). All the BrdU positive cells were counted blindly and manually using the ImageJ software. Results are shown as mean ±SEM of 10 animals and 4-6 slices per animal. Data was analyzed by Student's t test. *p<0.05 and ****p<0.0001 compared to controls

Because of the overall increase in hippocampal neuronal number in anti-aggregant Tau^RDΔKPP^ slices (Fig. 2a) we suspected an enhanced proliferation of hippocampal stem cells. Neurogenesis within the brain is demonstrated using an exogenous cell tracer, Bromodeoxy uridine (BrdU) which incorporates into the dividing cells during DNA synthesis. OHSCs are a suitable model to study postnatal neurogenesis [59]. BrdU labeling in OHSCs was done by adding freshly prepared 50μM BrdU in 1 ml of slice culture media. Since BrdU is incorporated into DNA during mitosis, its density decreases during successive cell divisions [20]. We therefore added 50μM BrdU into the slice culture media every second day after media exchange, thereby preventing the disappearance of highly proliferating cells as seen by BrdU immune-reactivity. The application of BrdU in the slice cultures began at DIV15 until DIV30. The OHSCs were immunostained for BrdU for proliferating cells at DIV30, revealing BrdU positive cells in the CA1, CA3, and DG (Fig. 6b). Such cells were found in controls, anti-aggregant and pro-aggregant slices and in all the regions of the hippocampus, consistent with reports showing that there could be a reorganization of the neurogenic niche in the OHSCs during the culturing period [37]. However, in case of the anti-aggregant Tau^RDΔKPP^ slices there was a remarkable 30% increase in the number of BrdU positive cells in the CA1 and CA3 regions and almost 100% increase in DG (Fig. 6b, bar 2, 5, and 8). This was in striking contrast to the pro-aggregant Tau^RDΔK^ slices where there was no significant difference in the number of BrdU positive cells compared to the age matched controls (Fig. 6b, bar 3, 6, and 9). Having confirmed the increase in proliferation in the anti-aggregant slices, we wanted to check whether the proliferating cells mature into neurons. BrdU was applied to the culture media from DIV15 until DIV 30, followed by double immunostaining with BrdU for the proliferating cells and NeuN for mature neurons. This showed that most of the cells were double-positive for BrdU and NeuN. The number of cells positive for BrdU increased by 90% in the CA1, 70% in CA3 and 100% in DG region of the anti-aggregant Tau^RDΔKPP^ slices (Additional file 2: Figure S1A, bars 3, 7 and 11). Out of these BrdU positive cells certain numbers of cells were co-labeled for NeuN. In particular in the CA3 and DG regions almost 70% and in CA1 almost 50% of the new born cells got differentiated into NeuN positive neurons in the anti-aggregant Tau^RDΔKPP^ slices (Additional file 2: Figure S1A, bars 4, 8, 12). This reveals an increase in proliferation followed by an increase in neuronal differentiation in the anti-aggregant Tau^RDΔKPP^ slices. By contrast, in the pro-aggregant Tau^RD∆K^ slices, the rate of proliferation in the CA3 and DG regions remained the same as seen in the controls (Additional file 2: Figure S1B, bars 5, 7, 9, 11). This also resulted in a 30% increase in the rate of differentiation in the CA1 region of the pro-aggregant Tau^RDΔK^ slices (Additional file 2: Fig. S1B, bars 2 and 4). This indicated that the expression of the pro-aggregant Tau^RD∆K^ does not have much influence on the proliferation and differentiation of the hippocampal stem cells.

### Switch-off of anti-aggregant Tau^RDΔKPP^ decreases neuronal number in slices

**Fig. 7:**
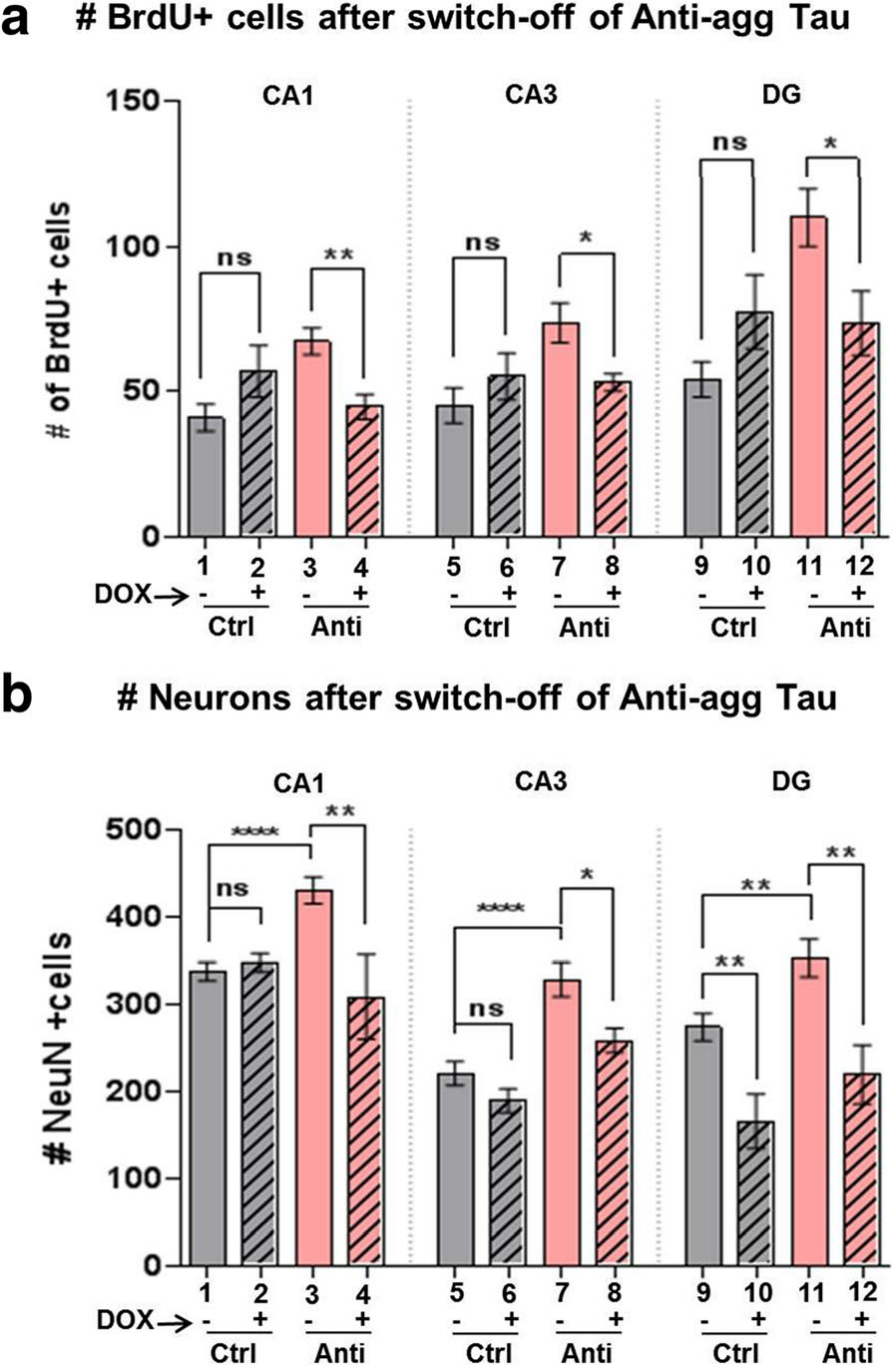
Switching-off the expression of mutant Tau. 2μM DOX was applied to the culture media of the slices to switch-off the expression of the exogenous human Tau constructs from DIV15 to DIV30. Bioluminescence was done after the DOX application on the slice cultures to make sure that there is no further expression of the anti-aggregant Tau^RDΔKPP^ during the subsequent culturing period. Results show the mean ±SEM of 10 animals, 4-6 slices per animal. Data was analyzed by Student's t test. *p<0.05 and **p<0.01 compared to switch-ON and switch-OFF conditions. **a** Graph representing the number of BrdU positive proliferating cells in the control and anti-aggregant Tau^RDΔKPP^ OHSCs before DOX application (switch-ON) and after (switch-OFF). There was a reduction of 32% in CA1, 27% in CA3 region and 33% in DG region in the number of BrdU positive cells in the DOX treated anti-aggregant Tau^RDΔKPP^ slices compared to the untreated anti-aggregant Tau^RDΔKPP^ slices (see pink open vs. hatched bars). This reduction parallels the decrease in the number of neurons in DOX-treated anti-aggregant Tau^RDΔKPP^ groups (see b). **b** Graph representing the number of NeuN positive neurons per counting frame, in the control and anti-aggregant Tau^RDΔKPP^ OHSCs before DOX application (switch-ON) and after (switch-OFF). There was a reduction of 21% in CA1, 22% in CA3 and 37% in DG region of the hippocampus, in the number of NeuN positive mature neurons in the DOX treated anti-aggregant Tau^RDΔKPP^ slices compared to the untreated anti-aggregant Tau^RDΔKPP^ slices (see pink open vs. hatched bars). Data was analyzed by Student's t test. *p<0.05, ** *p*<0.01 and ****p<0.0001 compared to controls

To prove that anti-aggregant Tau^RDΔKPP^ increases proliferation we further carried out switch-off experiments. OHSCs were treated with DOX from DIV15 to DIV30 and checked by bioluminescence for the decrease of expression of anti-aggregant Tau^RDΔKPP^. The slices did not show a bioluminescence signal after 6-8 hours of DOX application, verifying that expression had ceased. At DIV30 the slices were then fixed and checked for BrdU positive cells, number of neurons, and endogenous mouse Tau levels. Notably, after switch-off of anti-aggregant Tau^RDΔKPP^ there was a change in the number of BrdU stained cells in all regions of the hippocampus (CA1 region 32%, CA3 region 22% and DG 33% reduction) compared to switch-ON conditions (Fig. 7a, bars 4, 8 and 12). Similarly in the number of NeuN positive cells, there was a 22% reduction in CA1, 33% in CA3 and 37% reduction in DG compared to switch-On conditions (Fig. 7b, bars 4, 8 and 12).

### Enhanced post-natal hippocampal neurogenesis (at P8) in anti-aggregant Tau^RDΔKPP^ mice

**Fig. 8:**
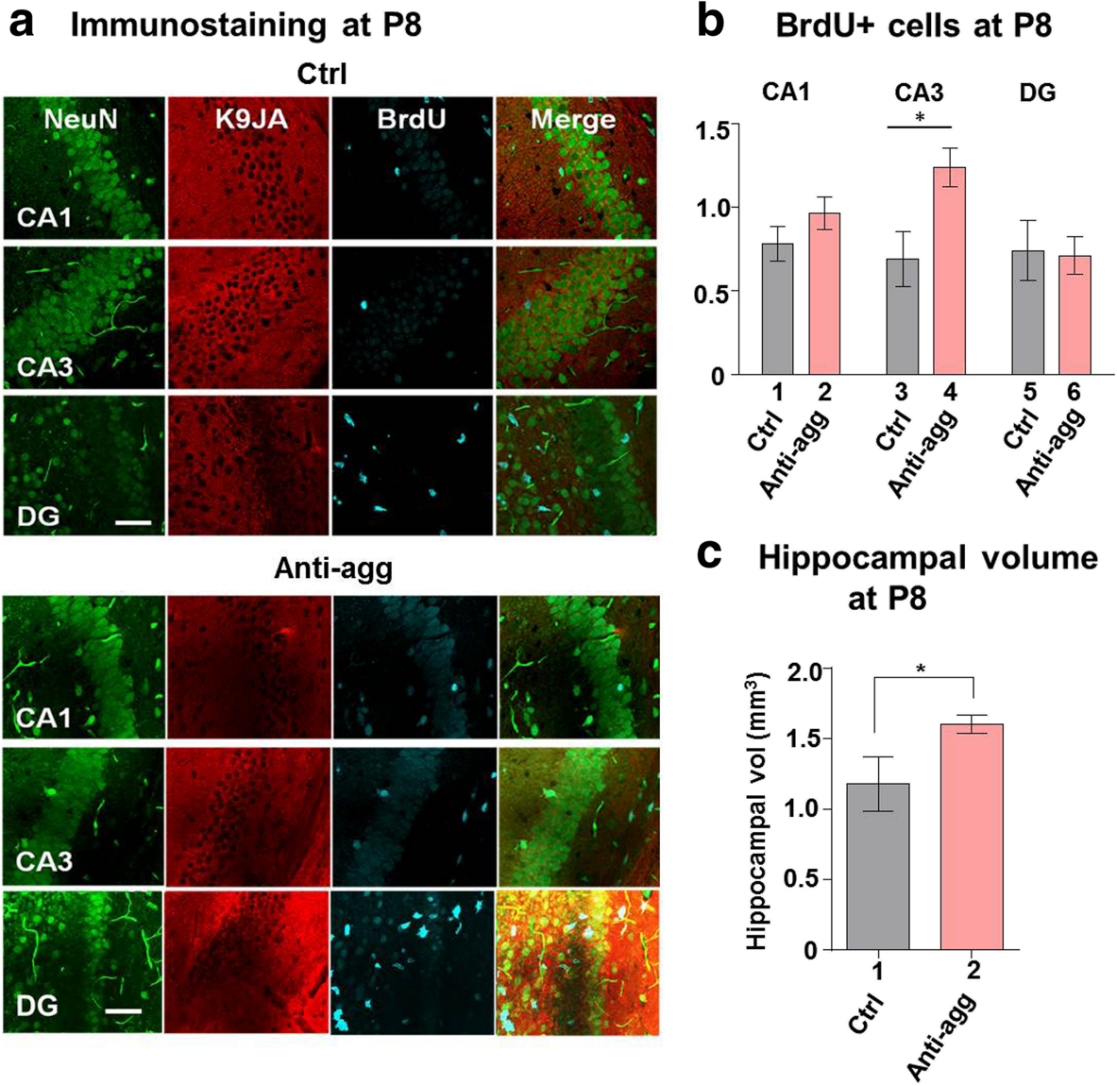
Proliferation of BrdU positive cells in P8 animals. P8 animals were given intra-peritoneal injections (i.p) of BrdU (50mg/kg body weight) 2 hours before sacrifice. The whole brain was cut into 30μM thick vibratome sections and slices were stained for neurons (NeuN), Tau (K9JA) and BrdU (BrdU antibody). **a** Representative images of NeuN (green), K9JA (red) and BrdU (cyan) staining in the CA1, CA3 and DG areas of the hippocampus of P8 control and anti-aggregant Tau^RD∆KPP^ mice. Scale bar 50μm. **b** Number of BrdU positive cells was counted by stereology. An increase (80%) was observed in the CA3 region in the anti-aggregant Tau^RDΔKPP^ pups compared to controls (compare bars 3, 4). Data were analyzed by Student's t test. *p<0.05 compared to controls. **c** Volume of the hippocampus in control and anti-aggregant Tau^RDΔKPP^ P8 pups. An increase in the volume of the hippocampus (25%) was observed in anti-aggregant Tau^RDΔKPP^ animals. The values determined are the apparent volumes of hippocampus. Results show the mean ±SEM (*n*= 3-5 animals/group) and data were analyzed by Student's t test. *p<0.05 compared to controls

The expression of the anti-aggregant Tau^RDΔKPP^ begins at an early embryonic stage of the mice. Since P8 animals were used for the preparation of OHSCs and since we observed drastic changes in hippocampal volume and neuronal number upon further ex-vivo cultivation, we analyzed these parameters in live animals at the post-natal age (P8). The animals were injected with BrdU and sacrificed after two hours. In this period BrdU becomes incorporated into the proliferating cells, but these cells have not yet migrated from their neuronal niche. This way we could analyze the number of proliferating cells and their localization in the hippocampus. To analyze post-natal neurogenesis, brain sections from P8 anti-aggregant Tau^RDΔKPP^ and control animals were immunostained for mature neurons (Fig. 8a, NeuN, green), proliferating cells (Fig. 8a, BrdU, cyan) and Tau (Fig. 8a, K9JA, red) and the BrdU positive cells were analyzed by stereology (Fig. 8b). BrdU positive cells were present in CA1, CA3 and DG in both the controls and anti-aggregant Tau^RDΔKPP^ groups (Fig. 8a, b), but their numbers were increased strongly by 80% only in the CA3 region of the anti-aggregant Tau^RDΔKPP^ mice (Fig. 8b, bar 4), with 20% change in the CA1 and no change in the DG (Fig. 8b, bar 2 and 6). Thus, apart from the well-known case of the DG, the CA3 might also have its stem cell niches maintained into adulthood. Additionally, there was an increase by 25% of the hippocampal volume in anti-aggregant Tau^RDΔKPP^ mice, compared to controls at P8 (Fig. 8c, bar 2), presumably due to the increased number of neurons.

**Fig. 9:**
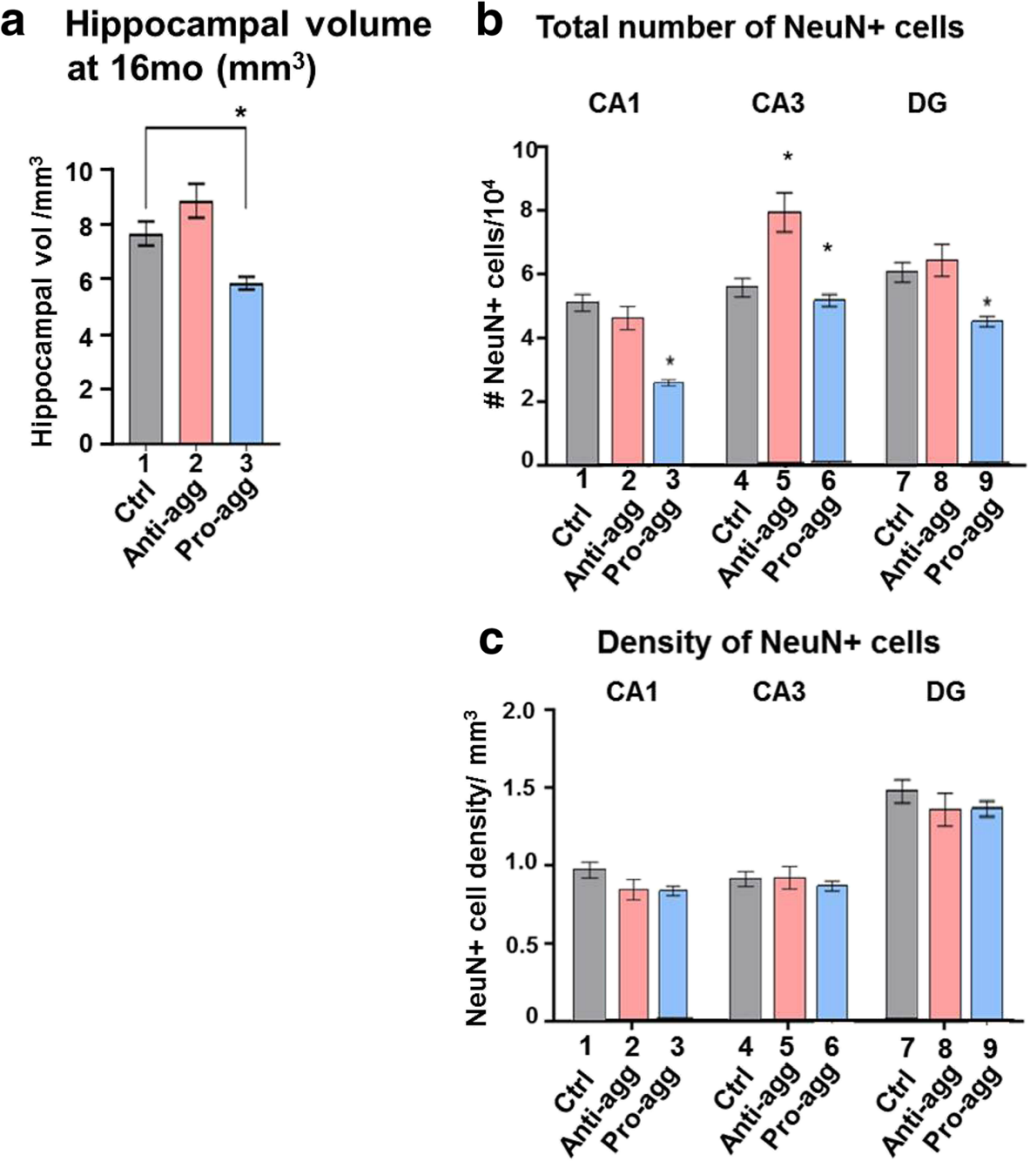
Analysis of hippocampal volume and neuronal numbers in old mice (16 months). **a** Stereological analysis of the apparent volume of the hippocampus, **b** number of NeuN positive neurons and c density of the neurons in the CA1, CA3 and DG regions of the hippocampus. The analysis was done on 16 month old animal brains from the control mice, anti-aggregant Tau^RDΔKPP^ mice, and pro-aggregant Tau^RDΔK^ mice. **a**, **b** There was an increase (~15%) of the hippocampal volume in the old-aged animals from the anti-aggregant Tau^RDΔKPP^ mice compared to the age-matched controls. This correlates with an increase of neurons (~20%) in the CA3 region, b, bar 5). By contrast, in pro-aggregant old mice the volume decreases 25% relative to controls (**a**, bar 3); this correlates well with the 10-50% loss of neurons in all regions (**b**, bars 3, 6, 9). **c** The density of neurons per mm^3^ was similar in controls, anti-aggregant and pro-aggregant old mice. Results represent the mean ±SEM (*n*= 3-5 animals/group)

We then asked whether the changes at embryonic states persisted throughout life and examined hippocampal volume and neuronal number in aged mice (16mo) (Fig. 9). The anti-aggregant Tau^RDΔKPP^ mice had an increased hippocampal volume of ~15% compared to age-matched controls but it was not a significant increase as analyzed by bonferroni post hoc test (Fig. 9a, bar 2) in contrast mice expressing pro-aggregant Tau^RDΔK^ had a 25% reduced hippocampal volume (Fig. 9a, bar 3). Surprisingly, the anti-aggregant Tau^RDΔKPP^ mice had an increased neuronal number significantly in the CA3 region (20%, Fig. 9b, bar 5), in contrast to the pro-aggregant Tau^RDΔK^ mice where neuronal loss (e.g. CA1 ~50%, CA3 ~10%, DG ~25%) was observed in all regions of the hippocampus (Fig. 9b, bar 3, 6, 9). In either case the neuronal density remained unchanged in all regions of the hippocampus (Fig. 9c) in both the anti-aggregant Tau^RDΔKPP^ and pro-aggregant Tau^RDΔK^ mice at 16 months of age.

### Anti-aggregant Tau^RDΔKPP^ correlates with decreased Wnt5a levels, leading to inhibition of the non-canonical Wnt signaling pathway

**Fig. 10:**
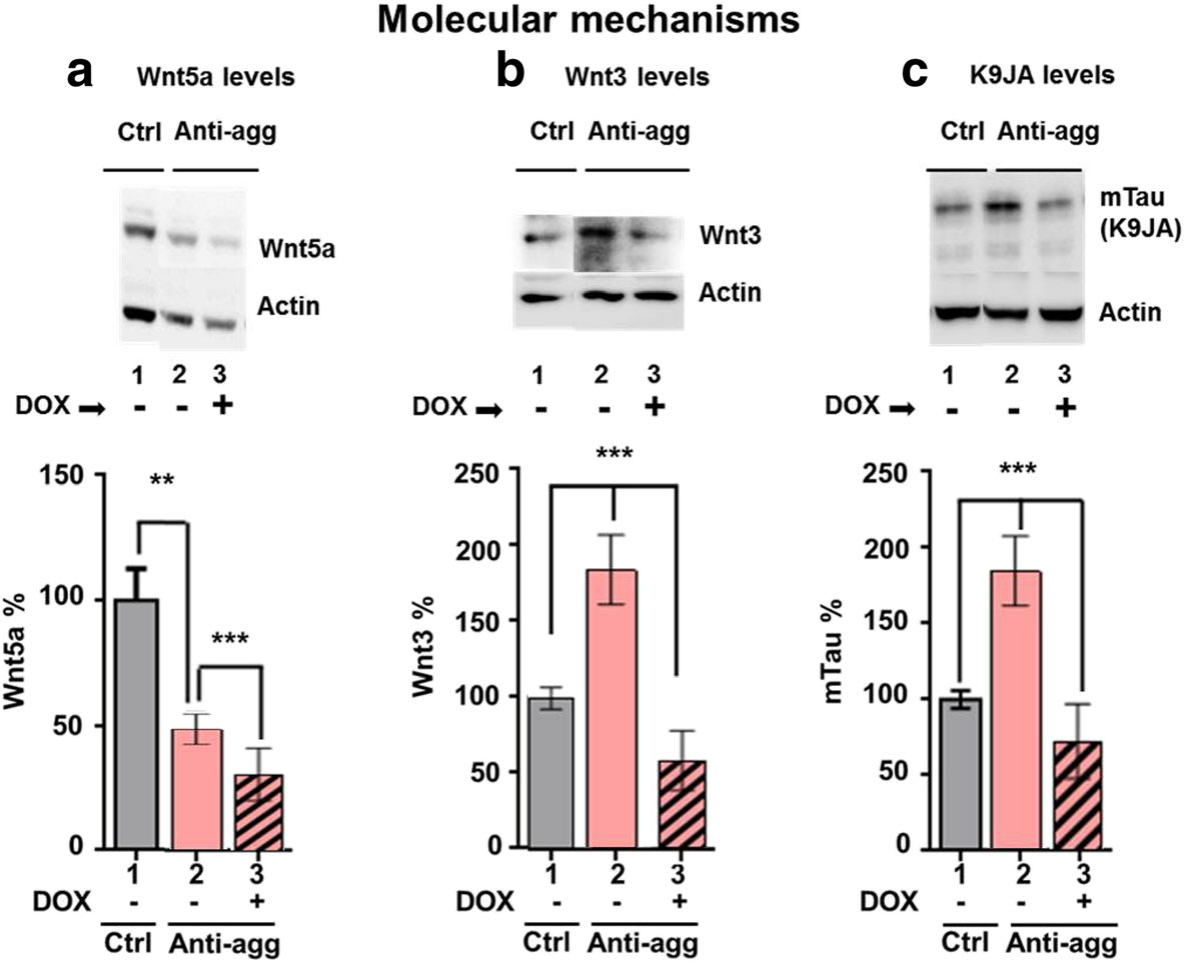
Identification of molecular signaling pathways affected by Tau. At DIV30, hippocampal tissue extracts from OHSCs of control and anti-aggregant Tau^RDΔKPP^ slices, without DOX (switch-ON) and with DOX (switch-OFF) were subjected to western blot analysis of Wnt5a, Wnt3, and Tau (K9JA). Results show the mean ±SEM of 4-6 slices per animal and 6-8 animals per condition and represent the ratio between the analyzed protein and actin levels. Quantification was obtained by densitometry and analyzed by Student's t test. **p<0.01 and ***p<0.001. **a** There was a significant decrease (50%) in the level of Wnt5a in the anti-aggregant Tau^RDΔKPP^ slices compared to the controls (bar 1, 2). When the expression of anti-aggregant Tau^RD∆KPP^ was switched off by addition of DOX from DIV15 to DIV30, the levels of Wnt5a in the anti-aggregant Tau^RDΔKPP^ slices were further reduced by 30% (bar 3). **b** There was an increase (85%) in the Wnt3 levels in the anti-aggregant Tau^RDΔKPP^ slice cultures compared to control slices (bar 1, 2). There was a pronounced reduction (40% below the control levels) when the expression of anti-aggregant Tau^RD∆KPP^ was switched off from DIV15 to DIV30 (bar 3). **c** There was 80% increase in the Tau levels as detected by the pan Tau antibody K9JA in the anti-aggregant Tau^RDΔKPP^ slice cultures (bar 2). When the expression of anti-aggregant Tau^RDΔKPP^ was switched off from DIV15 to DIV30 the Tau level was reduced by 40% to that in the controls (bar 3)

The Wnt signaling pathway is known to be involved in neurogenesis and neuroinflammation [35]. During pathological conditions like Parkinson Disease (PD) and AD there is deregulation of glial-neuron interactions which can be linked to the deregulation of the Wnt signaling pathway. Thus, the restoration of the Wnt signaling pathway is a promising approach in the field of neurodegeneration and aging [47]. Because of the relationships of Wnt5a signaling and neurogenesis we checked the level of Wnt5a in control and anti-aggregant Tau^RDΔKPP^ slices (Fig. 10a). OHSCs were cultured until DIV30 and doxycycline was added from DIV15 to DIV30 to some of the slices in order to switch-off the expression of the anti-aggregant Tau^RDΔKPP^. The OHSCs were then collected for biochemical analysis. Surprisingly there was a 50% reduction in the amount of Wnt5a protein in anti-aggregant Tau^RDΔKPP^ slices compared to controls (Fig. 10a, lane 2, bar 2). However, when the expression of the anti-aggregant Tau^RDΔKPP^ was switched off by DOX, there was an even more pronounced reduction in Wnt5a level (Fig. 10a, lane 3, bar 3). This indicates that the expression of the anti-aggregant Tau^RDΔKPP^ reduces the expression of Wnt5a irreversibly.

### Anti-aggregant Tau^RDΔKPP^ stimulates the canonical Wnt3 level

In aged hematopoietic stem cells the increase in Wnt5a levels acts as a molecular switch from canonical to non-canonical pathway, which thereby results in stem cell aging [25]. Wnt-β-catenin canonical signaling plays an important role in brain development and can modulate synaptic function [78]. Astrocytes play a major role in maintaining the subgranular zone (SGZ) and astrocyte-derived factors promote neurogenesis [5, 68], one of them being the canonical Wnt3 [51].

This prompted us to check whether such a molecular switch also exists in the mouse hippocampus. Hippocampal tissue lysates from DIV30 OHSCs were subjected to western blot analysis against Wnt3. Unexpectedly the Wnt3 levels in the anti-aggregant Tau^RDΔKPP^ slices were increased substantially (up to 85%) compared to the age-matched control slices (Fig. 10b, lane 2, bar 2). To confirm that this is due to the expression of the anti-aggregant Tau^RDΔKPP^, we applied DOX to the slice cultures from DIV15 until DIV30 to switch off the expression. Remarkably, this caused a 40% reduction of the Wnt3 level compared to control slices (Fig. 10b, lane 3, bar 3). This underscores the fact that the expression of anti-aggregant Tau^RDΔKPP^ causes a strong enhancement of Wnt3 levels. As mentioned above, anti-aggregant Tau^RDΔKPP^ slices had 80% more endogenous mouse Tau compared to the age matched control slices (Fig. 10c, lane 2, bar2). When the expression of anti-aggregant Tau^RDΔKPP^ was switched off by DOX, the endogenous mouse Tau was reduced to 20% below the control level (Fig. 10c, lane 3, bar 3). This suggests that the expression of anti-aggregant Tau^RDΔKPP^ is needed for the increased proliferation of newborn neurons, and since these new born neurons need endogenous mouse Tau for their migration, differentiation, and maturation, there is enhanced expression of endogenous mouse Tau. It has also been shown in previous studies that neuronal migration and maturation is inhibited in mouse brain after Tau reduction [66]. This data revealed that anti-aggregant Tau^RDΔKPP^ has an effect on proliferation, differentiation, and higher expression of endogenous mouse Tau.

### Hippocampal volume vs age

**Fig. 11:**
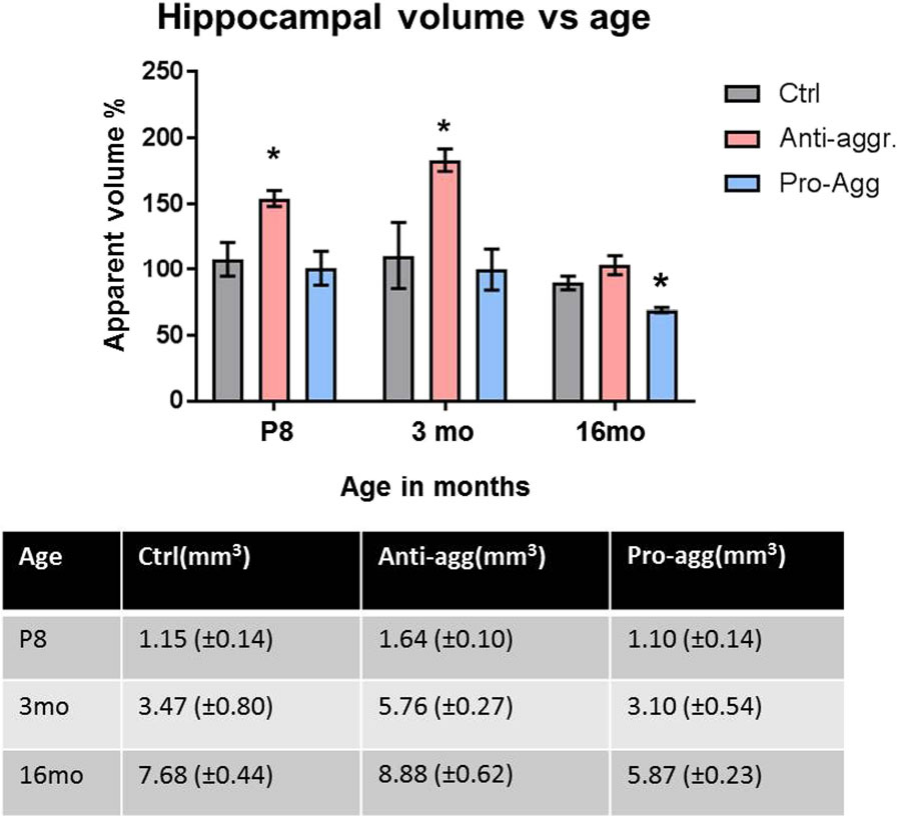
Hippocampal volume vs. age. Comparison of hippocampal volumes at P8, 3 months and 16 months of age, normalized to control =100% at each age (grey-control mice; pink- anti-aggregant mice and blue- pro-aggregant mice). The volumes of the anti-aggregant mice are higher than controls and those of the pro-aggregant mice are lower than the controls. Results represents the mean ±SEM (*n*= 3-5 animals/group) and data were analyzed by Student's t test. *p<0.05 compared to controls

The Fig. 11 illustrates the time course of changes in hippocampal volumes in the mice, normalized to the values of the controls at each age (=100%, grey bars). The values of the anti-aggregant mice lie consistently above those of the controls (pink bars), whereas pro-aggregant mice show smaller values (blue bars). The relative increase for the anti-aggregant mice are more pronounced at the earlier time points (P8 and 3 mo), in agreement with the enhanced neurogenesis. In case of the pro-aggregant mice the hippocampal volume decreases at advanced age due to the loss of neurons caused by the toxic Tau.

## Discussion

The background of this work is the earlier observation that expression of pro-aggregant forms of Tau cause the typical signs of tau pathology (e.g. hyperphosphorylation, aggregation, loss of synapses and neurons, cognitive deficits), whereas anti-aggregant forms do not [56, 71, 72]. These results strongly supported the notion that the aggregation of Tau (or at least its propensity for β-structure) is intimately linked to the disease process, and appeared to suggest that anti-aggregant Tau is only a neutral bystander in the functioning of neurons. However, the mechanisms of Tau-induced toxicity are still uncertain, and therefore we wanted to characterize mice expressing anti-aggregant Tau in more detail in order to understand the principal differences between the two forms of Tau. To this end we analyzed regulatable mice expressing anti-aggregant repeat domain (Tau^RDΔKPP^) and organotypic hippocampal slices (OHSCs) derived from them, and compared them with mice expressing the pro-aggregant repeat domain Tau^RDΔK^.

The OHSCs of mice expressing anti-aggregant Tau^RDΔKPP^ developed pronounced changes in brain structure (as observed at DIV30), starting with a massive increase in the overall hippocampal volume and number of neurons (by 50 to 80% in the CA1, CA3 and DG regions). By contrast, the OHSCs expressing the pro-aggregant Tau^RDΔK^ showed a pronounced loss of neurons (20 to 40 % in CA1, CA3 and DG). The increase in neuronal number in the anti-aggregant Tau^RDΔKPP^ OHSCs was the first indication that hippocampal neurogenesis could be enhanced even beyond the standard regions (subgranular zone).

It is known that adult hippocampal neurogenesis decreases with age in rodents and humans [45]. Contrary to expectations, the anti-aggregant Tau^RDΔKPP^ OHSCs revealed a ~100% increase in hippocampal stem cell proliferation in DG and ~30% increase in CA1 and CA3 as assessed by the BrdU incorporation assay. However, there was no change in BrdU positive proliferating cells in the pro-aggregant Tau^RDΔK^ OHSCs, compared to controls. This clearly shows that only the anti-aggregant Tau^RDΔKPP^ influences hippocampal stem cell proliferation whereas the pro-aggregant Tau^RDΔK^ does not. The enhanced proliferation in the anti-aggregant Tau^RDΔKPP^ OHSCs was followed by enhanced neuronal differentiation as assessed by DCX (Additional file 3: Figure S3) and Ki67 (Additional file 4: Figure S4) positive cells, resulting in an increase in NeuN positive mature neurons. Thus the expression of anti-aggregant Tau^RDΔKPP^ enhances the rate of proliferation as well as the rate of differentiation.

Having seen a massive increase in neuronal proliferation and differentiation in the anti-aggregant Tau^RDΔKPP^ OHSCs, we speculated whether there are changes in the overall expression and distribution of the endogenous mouse Tau, considering that Tau is involved in neuronal maturation and migration [66], and that the knock-down of Tau causes delay in stem cell maturation, retarded axonal and neuronal outgrowth [19]. Indeed the anti-aggregant Tau^RDΔKPP^ OHSCs showed an 80% increase of endogenous mouse tau which was not seen in the pro-aggregant Tau^RDΔK^ slices. Such an increase in endogenous mouse Tau would be expected to increase microtubule mass and stability in cells [38] and also to enhance outgrowth of cell processes and neurites in Sf9 and PC12 cells [43] and to promote neuronal morphogenesis [31, 42]. This increase in the endogenous mouse Tau in the anti-aggregant Tau^RDΔKPP^ OHSCs may be necessary for the morphogenesis of the newly developing neurons, thereby emphasizing the important role of Tau for normal differentiation and maturation of neurons. In other mouse models, an increase in endogenous Tau resulted in enhanced LTP in the DG region of the hippocampus and thereby improved memory (but without enhanced neurogenesis), consistent with improved reversal learning and fear conditioning [3, 9]. A similar effect was also observed in our anti-aggregant Tau^RDΔKPP^ mice that showed a robust increase of LTP in the CA1 region (see Fig. 10c in [72]).

When the expression of anti-aggregant Tau^RDΔKPP^ was switched-off from DIV15 to DIV30 in OHSCs, there was a reduction in the rate of proliferation (25% to 35% in CA1, CA3 and DG), differentiation (20% to 40% in CA1, CA3 and DG) compared to the switch-ON conditions, and a reduction of expression of endogenous mouse Tau (20%) compared to controls. This confirms that the expression of anti-aggregant Tau^RDΔKPP^ is coupled to an increased level of endogenous mouse Tau in a reversible manner.

The advantage of OHSCs is that the complexity of the brain structure is well maintained. Therefore this system can be used as an ex-vivo model for neuronal development and degradation [50]. Apart from neurons, non-neuronal cell types like astrocytes, microglia and oligodendrocytes are present and their origin and development can be studied [7, 53]. For example, with the expression of anti-aggregant Tau^RDΔKPP^ there was a dramatic reduction (~50%) in the number of microglia compared to controls. By contrast, in the pro-aggregant Tau^RDΔK^ slices there was a pronounced increase (100%) in the microglial number indicating massive neuroinflammation. Previous studies have shown that microglia are needed for (i) pruning of dendritic spines (ii) modulation of the pre- and postsynaptic structure of synapses on newborn neurons [60], (iii) maintenance of homeostasis of the neurogenic niche by the removal of newly born cells by apoptosis, and (iv) secretion of anti-inflammatory factors like IL10 and growth factors [63] which in turn regulate adult neurogenesis. Some studies have shown that the number of microglia are inversely correlated with the number of stem/progenitor cells, as shown in mice [70] and in co-culture experiments, despite the absence of inflammatory stimuli [61]. These studies match with our data regarding enhanced neurogenesis and reduced neuroinflammation in the anti-aggregant Tau^RDΔKPP^ OHSCs. Astrocytes exert profound effects on neuronal development as they provide support for neuronal survival, axon and dendritic outgrowth, neuritogenesis and synaptogenesis [17, 75]. In anti-aggregant Tau^RDΔKPP^ OHSCs the astrocytes were less hypertrophic, whilst in the control OHSCs they were hypertrophic as observed during normal aging. The GFAP level determined by western blotting in the hippocampal lysates from the control and anti-aggregant Tau^RDΔKPP^ OHSCs showed no changes in the level of GFAP. We further investigated the differentiation of NSCs into non-neuronal cell types like astrocytes and microglia in anti-aggregant Tau^RDΔKPP^ OHSCs and found that there were no cells double-positive for GFAP and BrdU, nor for Iba1and BrdU, indicating that there was no differentiation of the proliferating cells into astrocytes or microglia (data not shown). All this data indicates that with the expression of the anti-aggregant Tau^RDΔKPP^ the proliferation of hippocampal NSCs leads mainly to their differentiation into neurons.

Considering the increase in proliferation and neuronal numbers in anti-aggregant Tau^RDΔKPP^ OHSCs, we were interested to analyze whether these changes are restricted to the slice cultures, or whether they occurred also in postnatal animals. Anti-aggregant Tau^RDΔKPP^ mice were therefore analyzed at post-natal day 8 (P8) for BrdU positive proliferating cells. The post-natal neurogenesis, a transition state between embryonic and adult neurogenesis in the hippocampus, is important because it provides information about how neurogenesis continues into adulthood [58]. Previous reports had shown that during post-natal stages of brain development the hilus region of the hippocampus shows more neurogenesis [1] and more than half of the granule cells are born post-natally (P5-P14). We observed an increase of BrdU positive cells at P8 in anti-aggregant Tau^RDΔKPP^ pups. This was observed only in CA3 (80%), a region where enhanced proliferation of BrdU positive cells has not been reported so far. Consistent with this, the overall hippocampal volume in anti-aggregant Tau^RDΔKPP^ pups was increased as well (~25% at P8, compared to controls). This gain of volume persisted through adolescence (30% at 3 months) and even in adult life (15% at 16 months). By contrast, the hippocampal volume of pro-aggregant mice remained comparable to controls through adolescence (3 months), but then lagged behind controls in adult life (25% reduction at 16 months). This effect appears to be related mainly to the loss of neurons by neurodegeneration during adulthood, because the rate of neurogenesis remained comparable to that of controls.

Longitudinal changes in hippocampal volume in mice have been investigated by a number of authors, using both stereology and MRI methods [12, 30, 64]. Broadly speaking, there is a phase of rapid growth from birth to about 3-4 months, followed by an almost steady state for the following 20 months. There are modulations in the growth rates and magnitudes of the young vs. adult stages which depend on mouse strain, gender, and various genetic and other factors [54, 62]. The time course observed in our experiments is consistent with published data, assuming that the adult volume is reached at about 4 months, somewhat later than the 3-month time points chosen here, which explains the increase between 3 and 16 months. However, the important feature is the systematic difference between pro- and anti-aggregant mice, relative to controls. Volumes of pro-aggregant mice are lower than controls (because neuronal generation of neurons is offset by degenerative loss), volumes of anti-aggregant mice are higher (because of enhanced neurogenesis without enhanced degeneration) (Fig. 11). In organotypic slices at DIV30 the same features are found for neuronal numbers in all hippocampal subfields (Fig. 2), indicating that similar relationships between neurogenesis and degeneration are already operating at early time points. Since aging processes are ~5 times faster in slices than in animals [21, 23, 27, 55, 69], DIV30 would correspond roughly to an adult age (5 months). By comparison, advanced age in humans is associated with a gradual decrease in volume which is exacerbated in neurodegenerative diseases [4, 24]. This late phase is not pronounced in old mice because of their relatively short life span.

To pin down the molecular mechanisms resulting from the expression of the anti-aggregant Tau^RDΔKPP^, we investigated the role of the Wnt signaling pathways. Wnt proteins are principal regulators of adult hippocampal neurogenesis and play a role in adult hippocampal function [51]. Another hint was the increased LTP in the CA1 region of the hippocampus of anti-aggregant Tau^RDΔKPP^ mice [72], as Wnt signaling plays an important role in hippocampal LTP, neurogenesis and neuroinflammation [13, 36, 74]. Wnt5a protein is up-regulated in AD brains [49], an increased level of Wnt5a is a marker of aging, and increased Wnt5a is a direct inhibitor of the canonical Wnt signaling pathway [25]. In anti-aggregant mice there was a 50% drop in the Wnt5a protein level (compared to age-matched control slices). From this data we conclude that the expression of anti-aggregant Tau^RDΔKPP^ suppresses Wnt5a levels and thereby enhances neurogenesis and reduces neuroinflammation in this mouse model. This is consistent with studies suggesting that upregulation of Wnt5a signaling is involved in the cognitive decline associated with aging and also with the physiopathology of AD [6]. In the case of Wnt3 protein, its expression persists in the hippocampus and it is mainly released by astrocytes to regulate neurogenesis. Wnt3 acts via the canonical Wnt/β-catenin signaling pathway [18, 51] and the downstream targets are involved in promoting adult neurogenesis [46]. With the expression of the anti-aggregant Tau^RDΔKPP^ there is a clear upregulation of Wnt3 protein by 85%, concomitant with a 50% reduction of Wnt5a. A reversal of the Wnt3 upregulation is seen when the expression of the anti-aggregant Tau^RDΔKPP^ was switched off by the application of DOX (the levels are reduced by 40% compared to controls). By contrast, the down regulation of Wnt5a cannot be reversed by switching off Tau^RDΔKPP^ because DOX itself has a suppressing effect on Wnt5a.

## Conclusion

The data suggest that an increase of Tau in a non-aggregating fashion enhances neurogenesis via the canonical Wnt signaling pathway, without stimulating neuroinflammation.

## Additional files

Additional File 1: Figure S2. Total Tau and GSK3ß levels in the aged anti-aggregant mice. Hippocampal tissue lysates from 16month old control and anti-aggregant animals were subjected to western blot analysis for total Tau, active GSK3ß (phosphorylated at Y216) and total GSK3ß. There was no significant change, though there was a significant 50% increase in the total Tau levels. Results show the mean ±SEM of 6-7 animals per condition and represent the ratio between the analyzed protein and actin levels. Quantification was obtained by densitometry. (PDF 81 kb)

Additional file 2: Figure S1. Rate of proliferation and neuronal differentiation is higher in anti-aggregant Tau^RDΔKPP^ slices at DIV30. The rate of proliferation (as assessed by BrdU) is compared to the rate of neuronal differentiation. OHSCs from the controls, anti-aggregant Tau^RDΔKPP^ and pro-aggregant Tau^RDΔK^ pups were cultured until DIV30. BrdU was applied to the culture media from DIV15 until DIV30. Later the slices were fixed with 4% formaldehyde and immunostained for BrdU (for proliferating cells) and NeuN (for neurons). (A) Graph representing the number of proliferating cells (BrdU, empty bars) vs. the number of cells differentiated into neurons (BrdU+NeuN, hatched bars) in the controls (grey) and anti-aggregant Tau^RDΔKPP^ slices (pink) at DIV30. In the anti-aggregant Tau^RDΔKPP^ slices the number of BrdU positive cells increased by 90% in CA1, 70% in CA3 and 100% in DG (compare empty grey and pink bars 1 to 3, 5 to 7 and 9 to11). Almost 75% of the proliferating cells get differentiated into mature neurons both in the DG and the CA3 region and 50% of the cells in the CA1 differentiate into neurons (bars 3 to 4, 7 to 8 and 11 to 12). Data was analyzed by Student's t test. *p<0.05, ** *p*<0.01 and***p<0.001 compared to controls. (B) Graph representing the number of proliferating cells (BrdU, empty bars) vs. the number of cells differentiated into neurons (BrdU+NeuN, hatched bars) in the controls (grey) and the pro-aggregant Tau^RDΔK^ slices (blue) at DIV30. Data was analyzed by Student's t test. (PDF 130 kb)

Additional file 3: Figure S3. DCX positive cells are increased in the anti-aggregant slices at DIV30. OHSCs were cultured until DIV30. The slices were then fixed and immunostained with NeuN antibody for neurons and DCX antibody for progenitor cells. DCX positive cells appear in controls and in the anti-aggregant slices in all regions of the hippocampus. The figure shows the CA3 and DG regions from the control and anti-aggregant slices at DIV30. Arrows indicate DCX+ cell bodies. Note the increase in DCX+ positive cells in the anti-aggregant Tau^RDΔKPP^ slices compared to the age-matched controls. (PDF 183 kb)

Additional file 4: Figure S4. Ki67 positive cells are increased in anti-aggregant slices at DIV30. OHSCs were cultured until DIV30. The slices were then fixed and immunostained with NeuN antibody for neurons and Ki67, a marker of proliferating cells. Note that with advanced age the level of proliferation is higher in anti-aggregant Tau^RDΔKPP^ slices (top row), compared with controls (bottom). (PDF 113 kb)

## Abbreviations

Aß: Amyloid beta
AD: Alzheimer disease
BrdU: Bromodeoxy uridine
CSF: Cerebrospinal fluid
CA1: Cornu ammonis 1
CA3: Cornu ammonis 3
CaMKIIα: Calcium/calmodulin-dependent protein kinase IIα
C-terminal: Carboxy-terminus
DG: Dentate gyrus
DIV: Days in vitro
DOX: Doxycycline
PD: Parkinson disease
FTD: Frontotemporal dementia
FTDP-17: Frontotemporal dementia and Parkinsonism linked to chromosome17
GFAP: Glial fibrillary acidic protein
LUC: Firefly luciferase
NFT: Neurofibrillary tangles
NSCs: Neural stem cells
NeuN: Neuronal nuclear protein
OHSCs: Organotypic hippocampal slice cultures
P8: Post-natal day 8
tTa: Tetracycline transactivator protein
Tau^RDΔK^: Tau Repeat Domain with FTDP-17 mutation ΔK280 (pro-aggregant)
Tau^RDΔKPP^: Tau Repeat Domain with FTDP-17 mutation ΔK280 plus two proline mutations (anti-aggregant)
