# Supplementary figures and images for: Anti-aggregant tau mutant promotes neurogenesis
Source: Mol Neurodegener. 2017 Dec 4;12:88. doi: 10.1186/s13024-017-0230-8 (PMC5715613; doi:10.1186/s13024-017-0230-8)

Sup Fig 2: Molecular pathways activated in 16 month old mice

A)

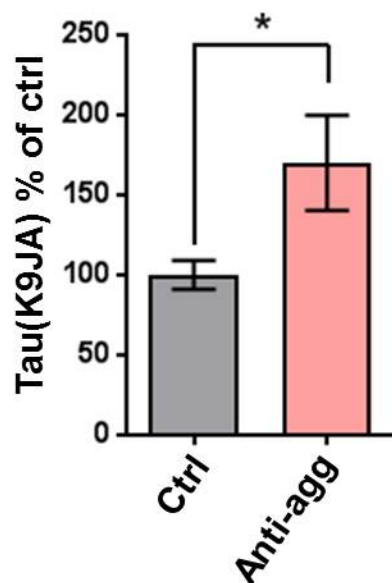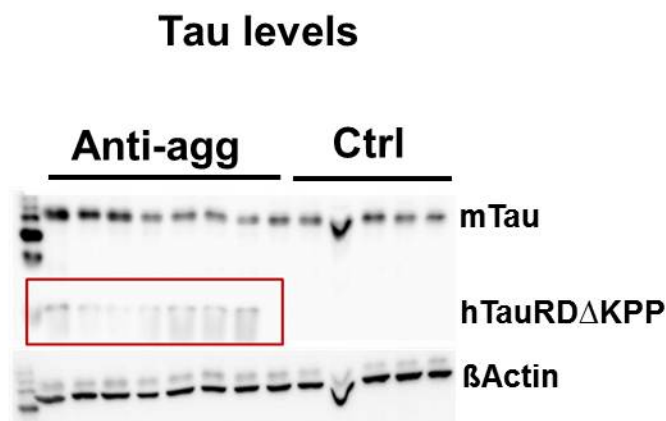

B)

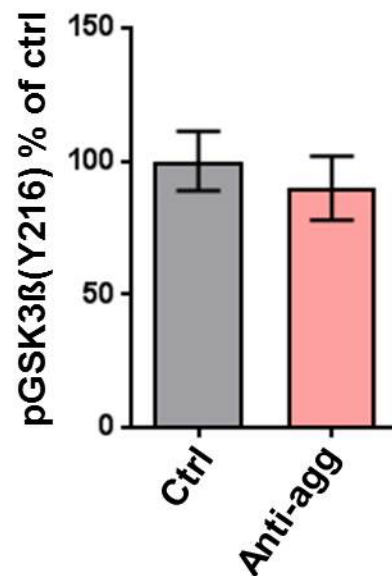

Active GSK3β levels

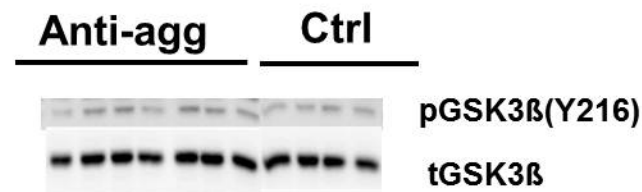

Supplement: Supplementary file 1 — Total Tau and GSK3ß levels in the aged anti-aggregant mice. Hippocampal tissue lysates from 16month old control and anti-aggregant animals were subjected to western blot analysis for total Tau, active GSK3ß (phosphorylated at Y216) and total GSK3ß. There was no significant change, though there was a significant 50% increase in the total Tau levels. Results show the mean ±SEM of 6-7 animals per condition and represent the ratio between the analyzed protein and actin levels. Quantification was obtained by densitometry. (PDF 81 kb) [file 13024_2017_230_MOESM1_ESM.pdf]

**Sup Fig 3: DCX+ cells at DIV30 (Ctrl and Anti-agg slices)**

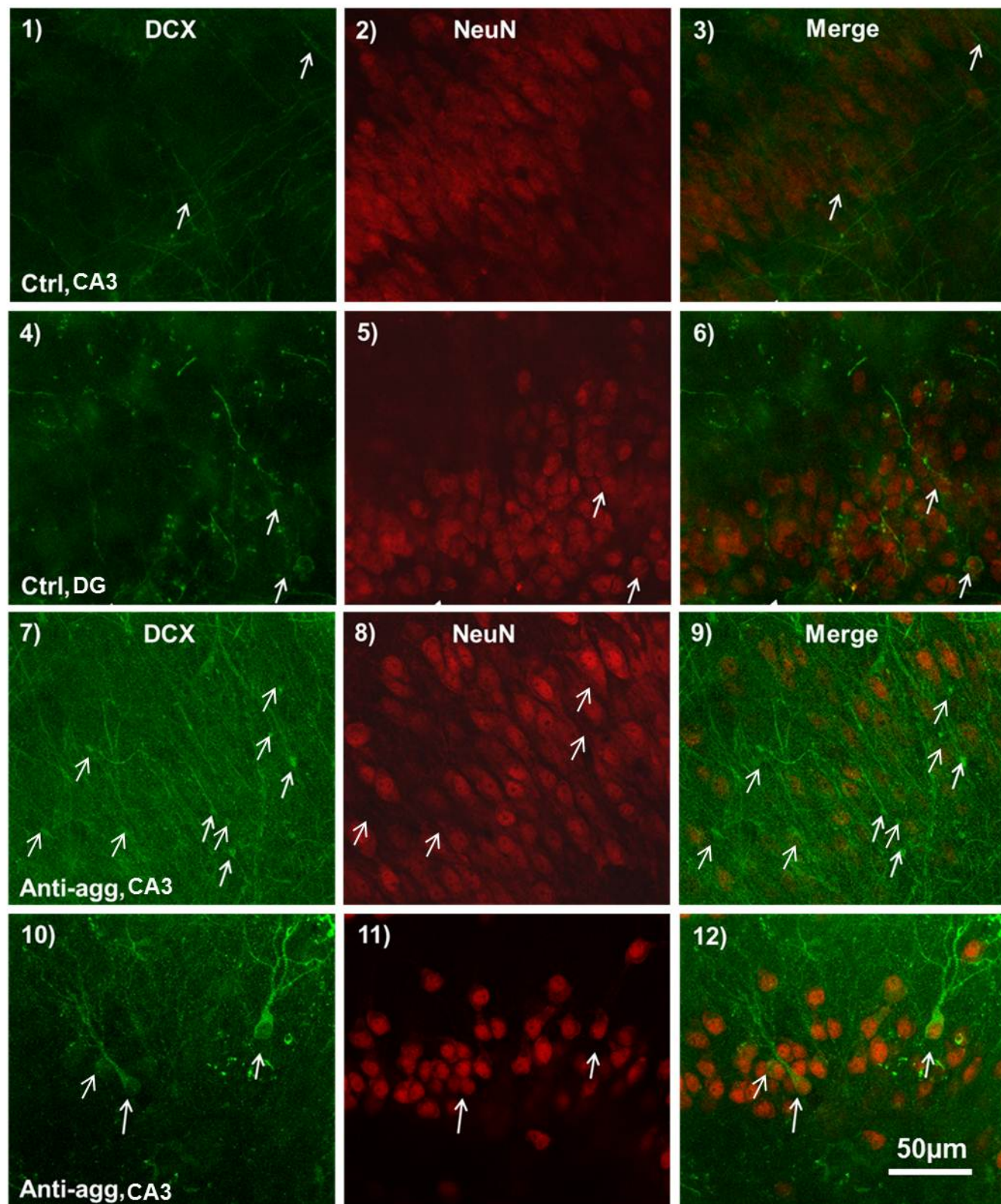

Supplement: Supplementary file 3 — DCX positive cells are increased in the anti-aggregant slices at DIV30. OHSCs were cultured until DIV30. The slices were then fixed and immunostained with NeuN antibody for neurons and DCX antibody for progenitor cells. DCX positive cells appear in controls and in the anti-aggregant slices in all regions of the hippocampus. The figure shows the CA3 and DG regions from the control and anti-aggregant slices at DIV30. Arrows indicate DCX+ cell bodies. Note the increase in DCX+ positive cells in the anti-aggregant TauRDΔKPP slices compared to the age-matched controls. (PDF 183 kb) [file 13024_2017_230_MOESM3_ESM.pdf]

**Sup Fig 4: Ki67 positive cells at DIV30 in Anti-agg slices**

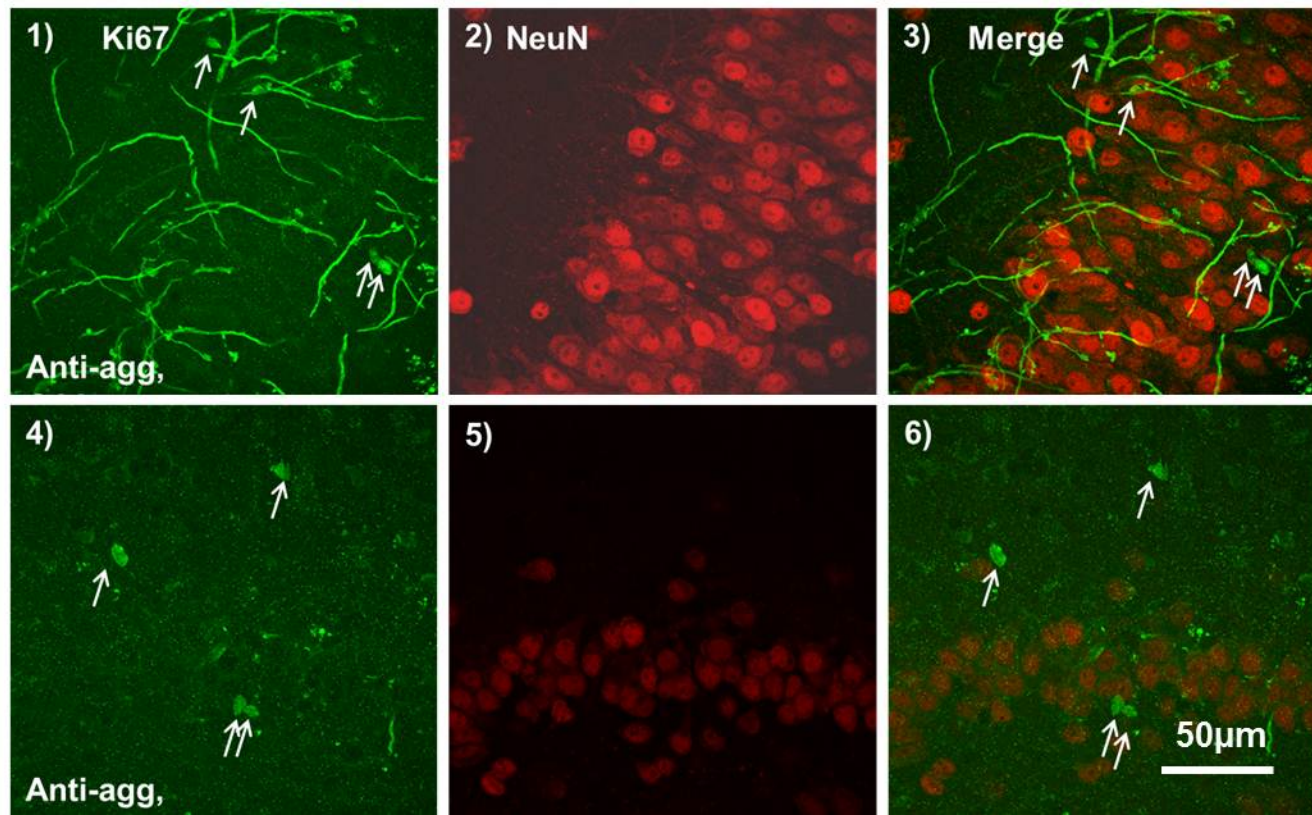

Supplement: Supplementary file 4 — Ki67 positive cells are increased in anti-aggregant slices at DIV30. OHSCs were cultured until DIV30. The slices were then fixed and immunostained with NeuN antibody for neurons and Ki67, a marker of proliferating cells. Note that with advanced age the level of proliferation is higher in anti-aggregant TauRDΔKPP slices (top row), compared with controls (bottom). (PDF 113 kb) [file 13024_2017_230_MOESM4_ESM.pdf]
