# Supplementary material for: Anti-aggregant tau mutant promotes neurogenesis
Source: Mol Neurodegener. 2017 Dec 4;12:88. doi: 10.1186/s13024-017-0230-8 (PMC5715613; doi:10.1186/s13024-017-0230-8)

Sup Fig 1:

A) BrdU and NeuN positive cells in Anti-agg slices at DIV30

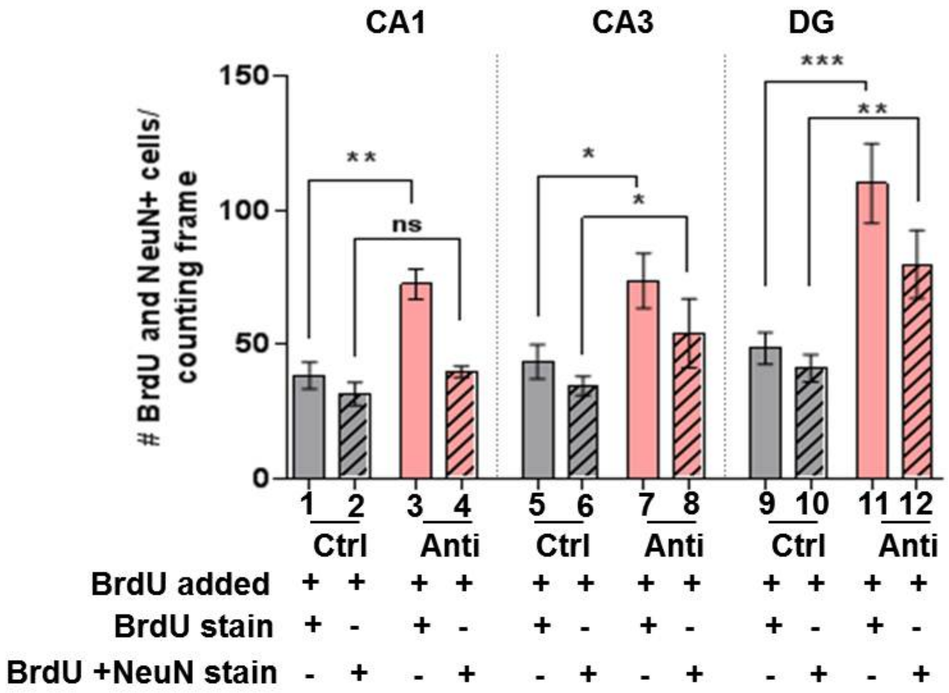

B) BrdU and NeuN positive cells in Pro-agg slices at DIV30

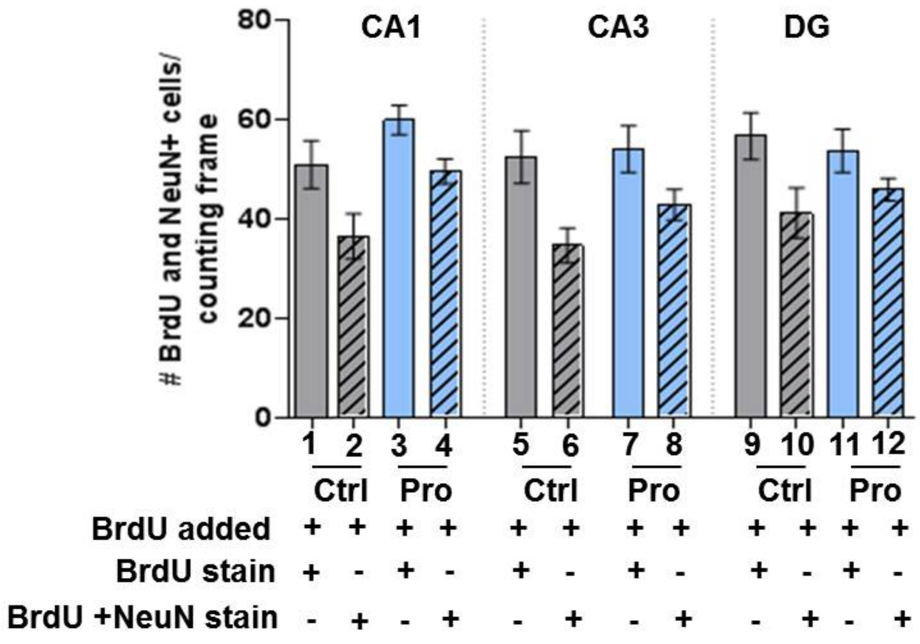

Supplement: Supplementary file 2 — Rate of proliferation and neuronal differentiation is higher in anti-aggregant TauRDΔKPP slices at DIV30. The rate of proliferation (as assessed by BrdU) is compared to the rate of neuronal differentiation. OHSCs from the controls, anti-aggregant TauRDΔKPP and pro-aggregant TauRDΔK pups were cultured until DIV30. BrdU was applied to the culture media from DIV15 until DIV30. Later the slices were fixed with 4% formaldehyde and immunostained for BrdU (for proliferating cells) and NeuN (for neurons). (A) Graph representing the number of proliferating cells (BrdU, empty bars) vs. the number of cells differentiated into neurons (BrdU+NeuN, hatched bars) in the controls (grey) and anti-aggregant TauRDΔKPP slices (pink) at DIV30. In the anti-aggregant TauRDΔKPP slices the number of BrdU positive cells increased by 90% in CA1, 70% in CA3 and 100% in DG (compare empty grey and pink bars 1 to 3, 5 to 7 and 9 to11). Almost 75% of the proliferating cells get differentiated into mature neurons both in the DG and the CA3 region and 50% of the cells in the CA1 differentiate into neurons (bars 3 to 4, 7 to 8 and 11 to 12). Data was analyzed by Student's t test. *p<0.05, ** p<0.01 and***p<0.001 compared to controls. (B) Graph representing the number of proliferating cells (BrdU, empty bars) vs. the number of cells differentiated into neurons (BrdU+NeuN, hatched bars) in the controls (grey) and the pro-aggregant TauRDΔK slices (blue) at DIV30. Data was analyzed by Student's t test. (PDF 130 kb) [file 13024_2017_230_MOESM2_ESM.pdf]
